# Supplementary material for: Generating functional and multistate proteins with a multimodal diffusion transformer
Source: bioRxiv. 2025 Sep 4:2025.09.03.672144. Preprint. [Version 1] doi: 10.1101/2025.09.03.672144 (PMC12424740; doi:10.1101/2025.09.03.672144)
Supplement: 1 [file NIHPP2025.09.03.672144v1-supplement-1.pdf]

bioRxiv preprint doi: https://doi.org/10.1101/2025.09.03.672144; this version posted September 4, 2025. The copyright holder for this preprint (which was not certified by peer review) is the author/funder, who has granted bioRxiv a license to display the preprint in perpetuity. It is made available under aCC-BY 4.0 International license.

B Supplementary Results

Figs. S1, S2, S3 show additional results, broken down by protein length, on the success rates, pLDDT, and scTM of ProDiT and baselines in unconditional sequence generation, unconditional structure generation, and co-generation, respectively. Fig. S4 shows similar results for structure generation and co-generation by scRMSD.

Fig. S5 compares the scTM and scRMSD of unconditional structure generation with ProDiT when inverse folding with 8x ProteinMPNN sequences versus inverse folding with ProDiT. We note that conditional generations with GO terms use ProDiT for inverse folding. Due to degraded performance, we also explore structure generation with $\nu = 0$, which produces more designable structures. We use $\nu = 0$ and inverse folding with ProDiT for GO term conditioning.

Figs. S6, S7, and S8 show diversity metrics, broken down by protein length, for sequence generation, structure generation, and co-generation respectively. In particular, TM-diversity (all) pools together all pairwise TM-scores within generations of the same length, i.e., the violinplot is a density estimator over 9900 TM-scores per protein length. On the other hand, TM-diversity (max) assigns $\max_{i \neq j} TM_{i,j}$ to be the diversity of generation i. All TM-scores are computed with TMalign.

All violin plots show the mean and inter-quartile range. Unless otherwise noted, all metrics and evaluation procedures follow their definitions in the main text.

Figs. S9 and S10 show statistics from the structural alignment pipeline for function-conditioned design.

Figs. S11 and S12 show the full lysozyme active site motif and the individual structure predictions for the selected scaffold in the bound and unbound state, along with full motif RMSDs. Figs. S13 and S14 show similar structures for the lysozyme active site motif and scaffold.

Table S1 lists the success rates, scTM, TM-diversity, and TM-novelty (when available) for all 915 GO terms evaluated for function conditioning. Table S2 provides additional results for 45 GO terms with successful structural alignment hits.

bioRxiv preprint doi: https://doi.org/10.1101/2025.09.03.672144; this version posted September 4, 2025. The copyright holder for this preprint (which was not certified by peer review) is the author/funder, who has granted bioRxiv a license to display the preprint in perpetuity. It is made available under aCC-BY 4.0 International license.

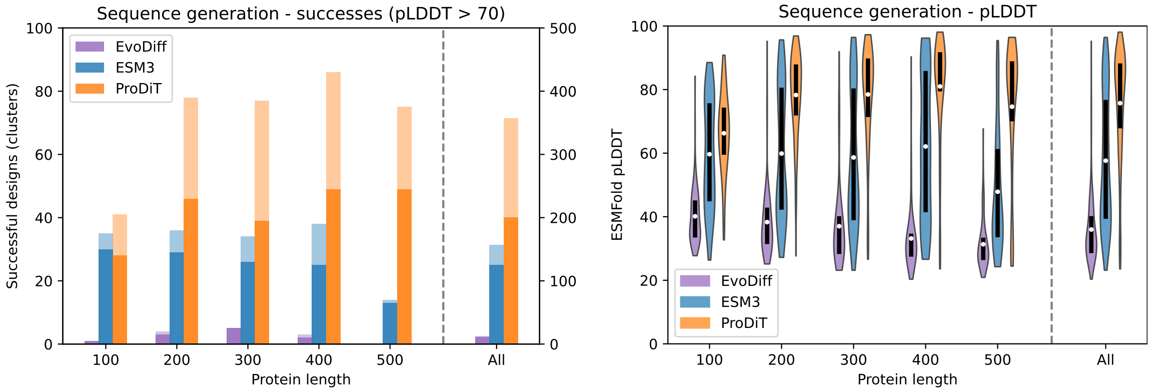

Figure S1: Success rates and ESMFold pLDDT for unconditional sequence generation.

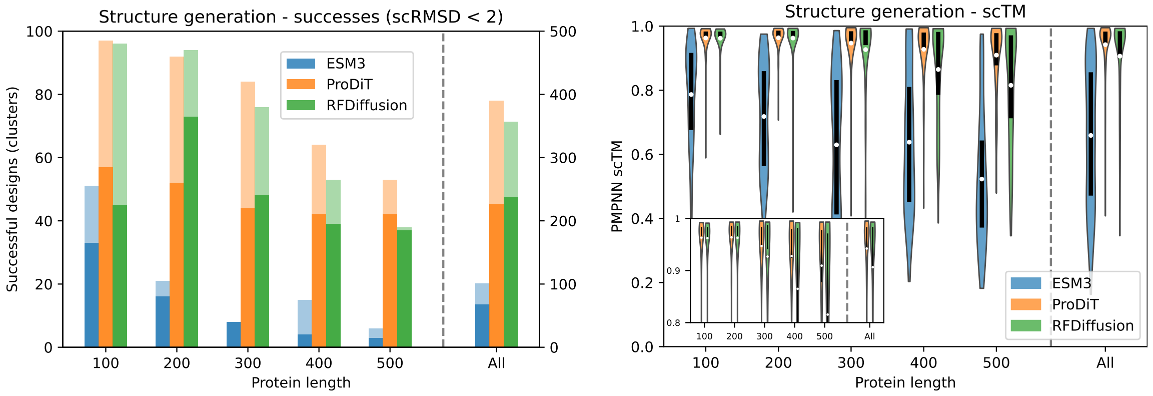

Figure S2: Success rates and scTM for unconditional structure generation.

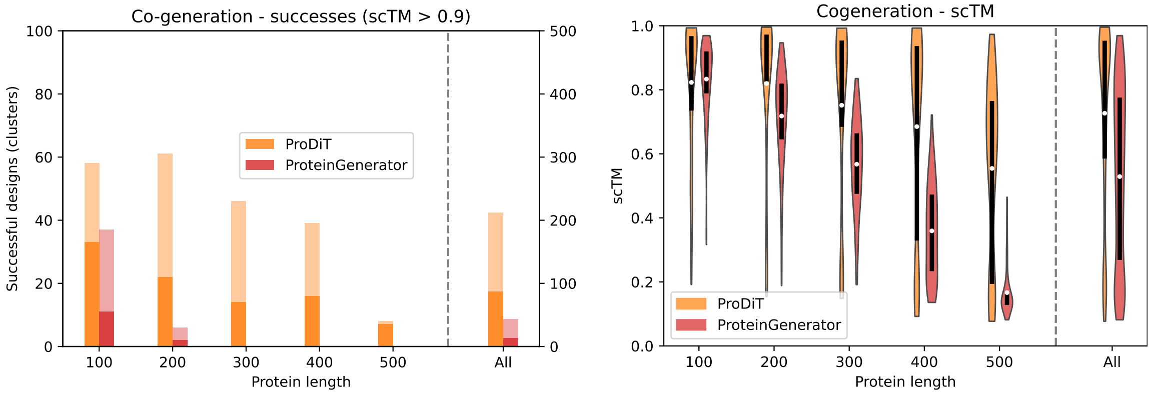

Figure S3: Success rates and scTM for sequence-structure co-generation.

bioRxiv preprint doi: https://doi.org/10.1101/2025.09.03.672144; this version posted September 4, 2025. The copyright holder for this preprint (which was not certified by peer review) is the author/funder, who has granted bioRxiv a license to display the preprint in perpetuity. It is made available under aCC-BY 4.0 International license.

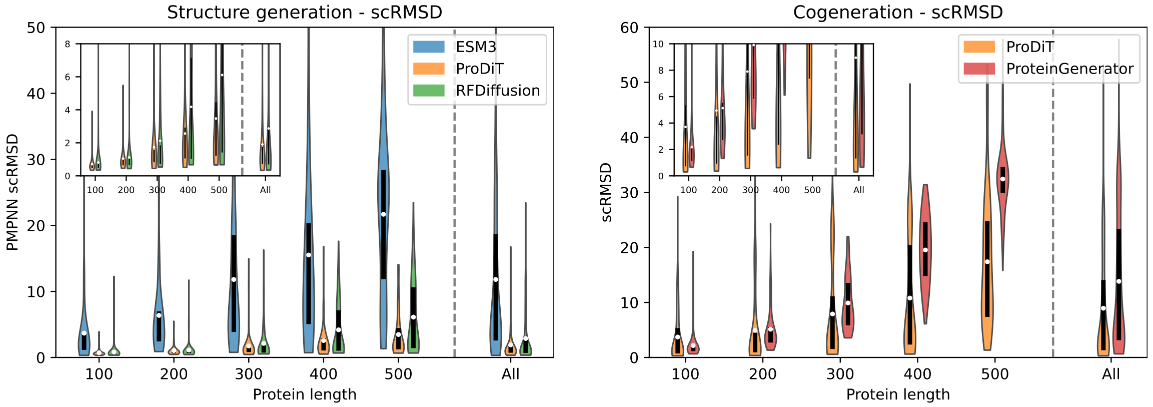

Figure S4: scRMSD for unconditional structure generation and sequence-structure co-generation

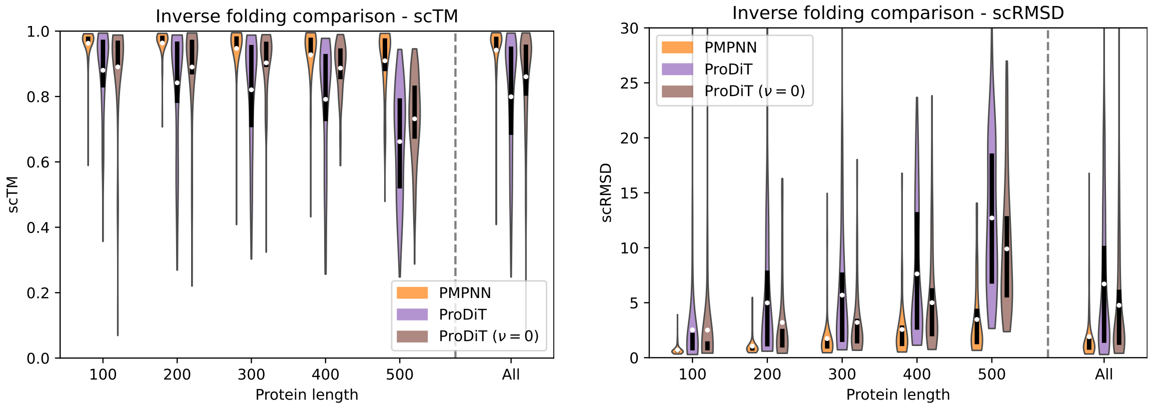

Figure S5: Comparison of inverse folding with ProteinMPNN vs ProDiT in structure generation.

bioRxiv preprint doi: https://doi.org/10.1101/2025.09.03.672144; this version posted September 4, 2025. The copyright holder for this preprint (which was not certified by peer review) is the author/funder, who has granted bioRxiv a license to display the preprint in perpetuity. It is made available under aCC-BY 4.0 International license.

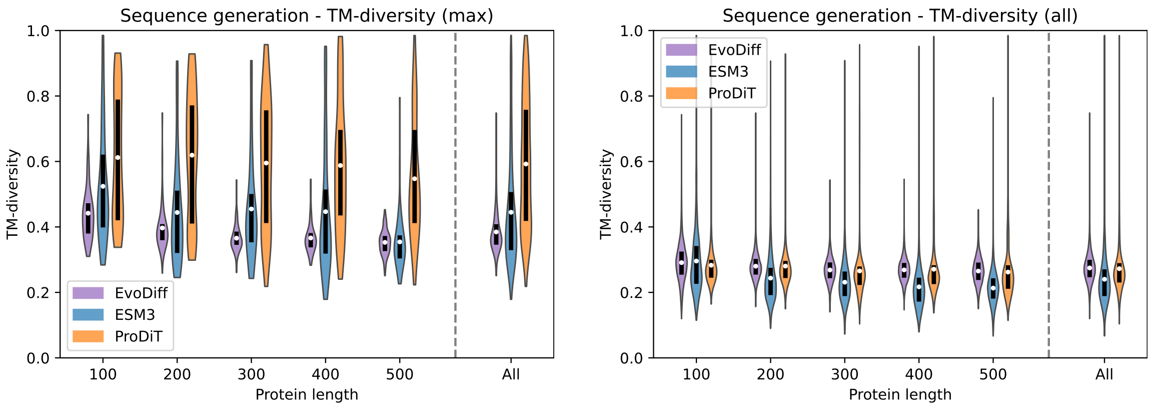

Figure S6: Diversity metrics for sequence generation. Lower TM-score means higher diversity.

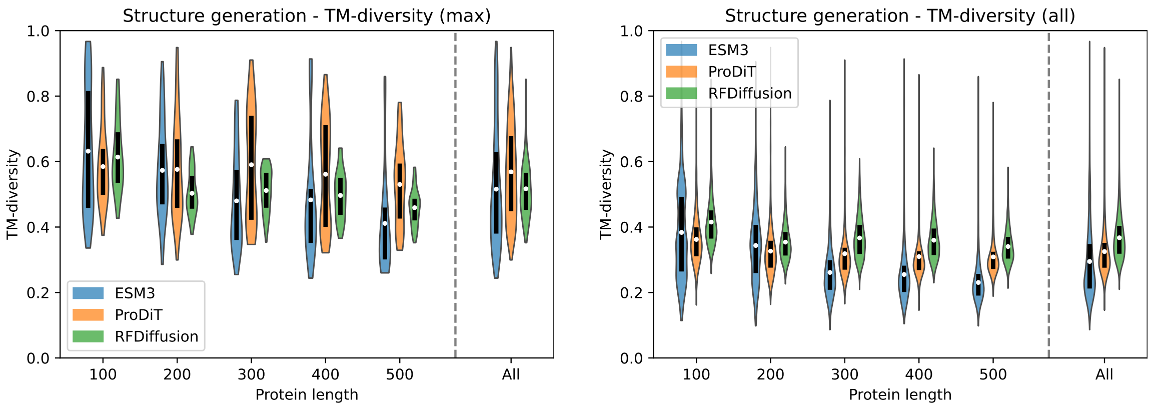

Figure S7: Diversity metrics for structure generation. Lower TM-score means higher diversity.

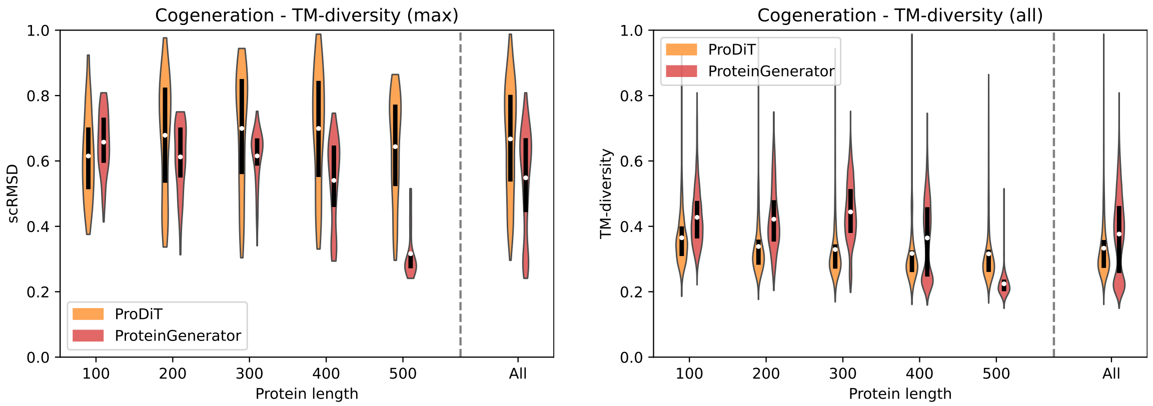

Figure S8: Diversity metrics for co-generation. Lower TM-score means higher diversity.

bioRxiv preprint doi: https://doi.org/10.1101/2025.09.03.672144; this version posted September 4, 2025. The copyright holder for this preprint (which was not certified by peer review) is the author/funder, who has granted bioRxiv a license to display the preprint in perpetuity. It is made available under aCC-BY 4.0 International license.

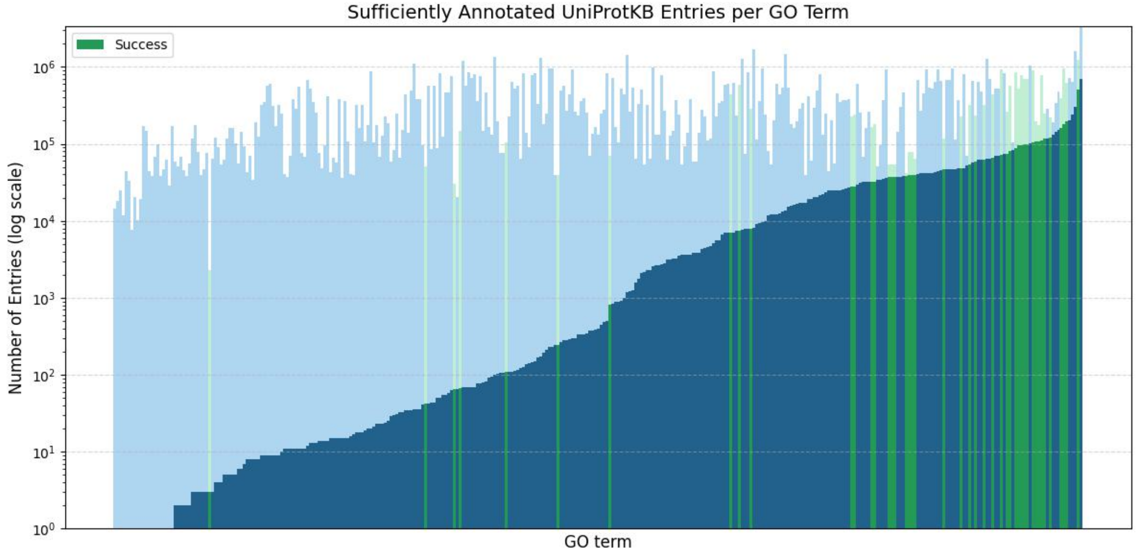

Figure S9: **GO terms by number of sufficiently annotated UniProtKB entries**, i.e., active sites with $\geq$2 residues. 337 GO terms are analyzed in total, out of 465 with successful ProDiT designs. Across GO terms, the median percentage of sufficiently annotated entries is 0.23%. 10 terms have zero annotated entries, and 132 terms have $<$100 annotated entries. Light bars indicate the number of total entries. GO terms with successful hits in our structural alignment pipeline (Methods) are highlighted in green.

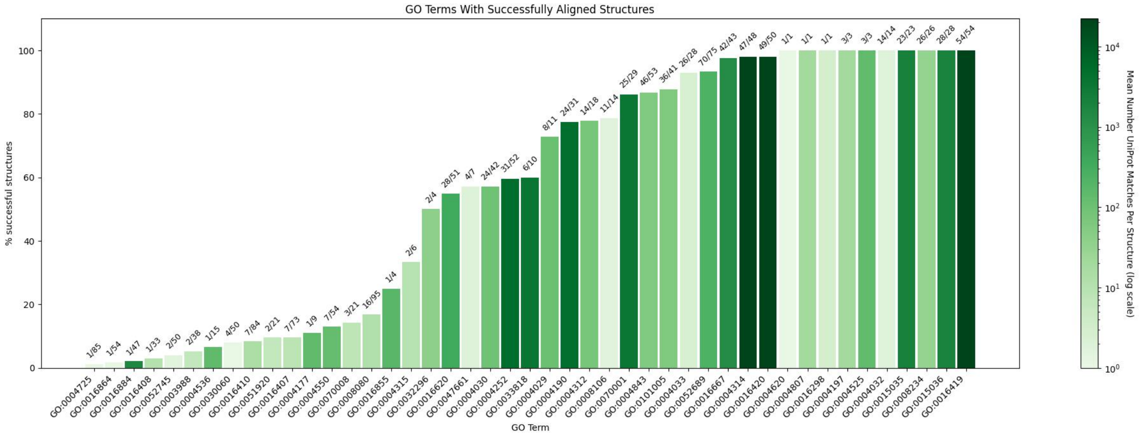

Figure S10: **Statistics of GO terms with successful hits from the structural alignment pipeline** (45 terms). Bars are labeled with the number of successfully aligned ProDiT generations out of total successful ProDiT generations, with the bar height indicating the percentage. The bar color indicates the mean number of matching UniProtKB entries per successful design. While most GO terms matched with a few entries (median 19), a few terms exceeded 20,000 aligned hits per design.

bioRxiv preprint doi: https://doi.org/10.1101/2025.09.03.672144; this version posted September 4, 2025. The copyright holder for this preprint (which was not certified by peer review) is the author/funder, who has granted bioRxiv a license to display the preprint in perpetuity. It is made available under aCC-BY 4.0 International license.

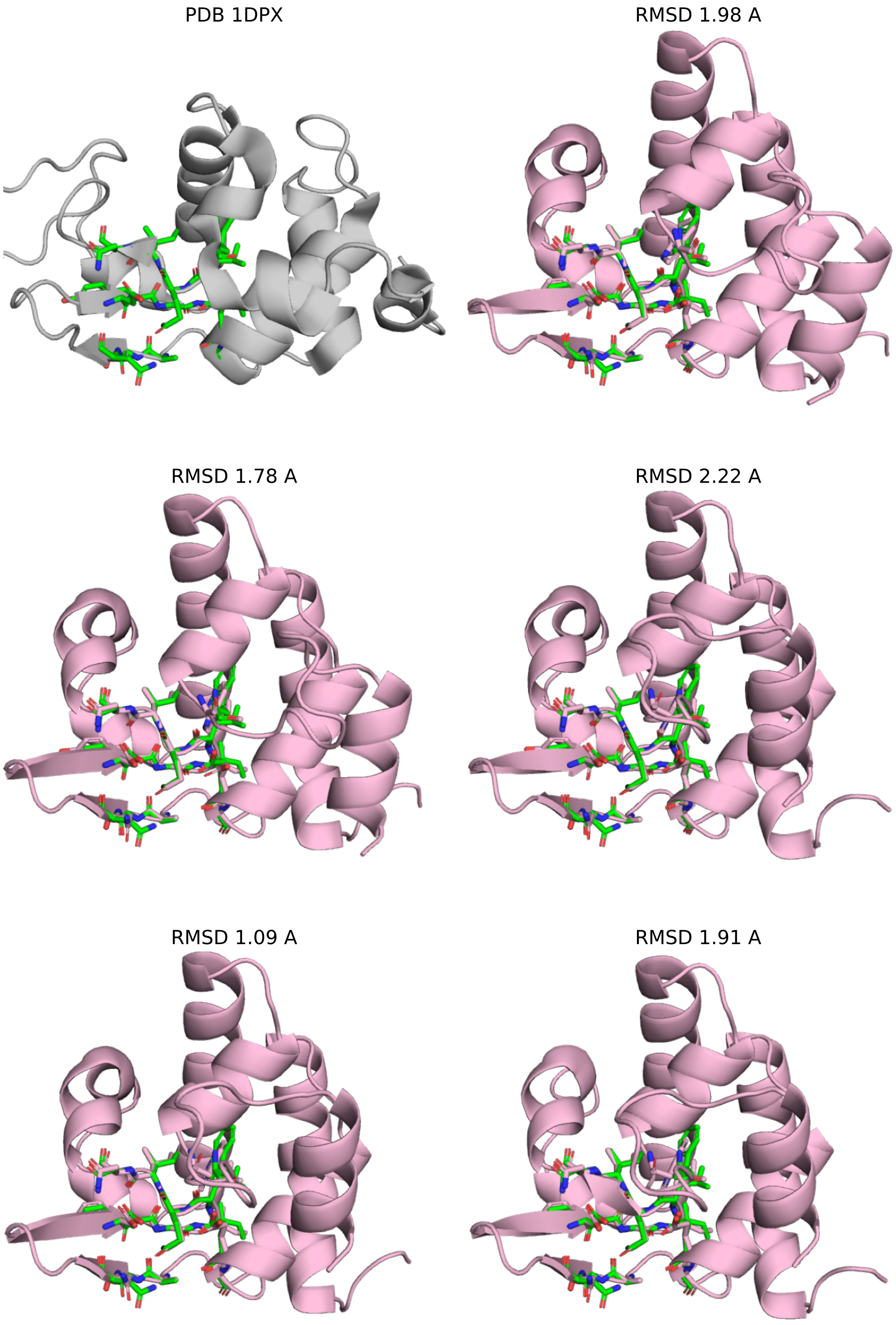

Figure S11: **Unbound states of the lysozyme motif scaffold**. Active site motif of lysozyme shown within PDB 1DPX (top left). The remaining structures show the five Chai-1 structure predictions without the calcium effector. The RMSD Cα RMSD across all motif residues is listed.

bioRxiv preprint doi: https://doi.org/10.1101/2025.09.03.672144; this version posted September 4, 2025. The copyright holder for this preprint (which was not certified by peer review) is the author/funder, who has granted bioRxiv a license to display the preprint in perpetuity. It is made available under aCC-BY 4.0 International license.

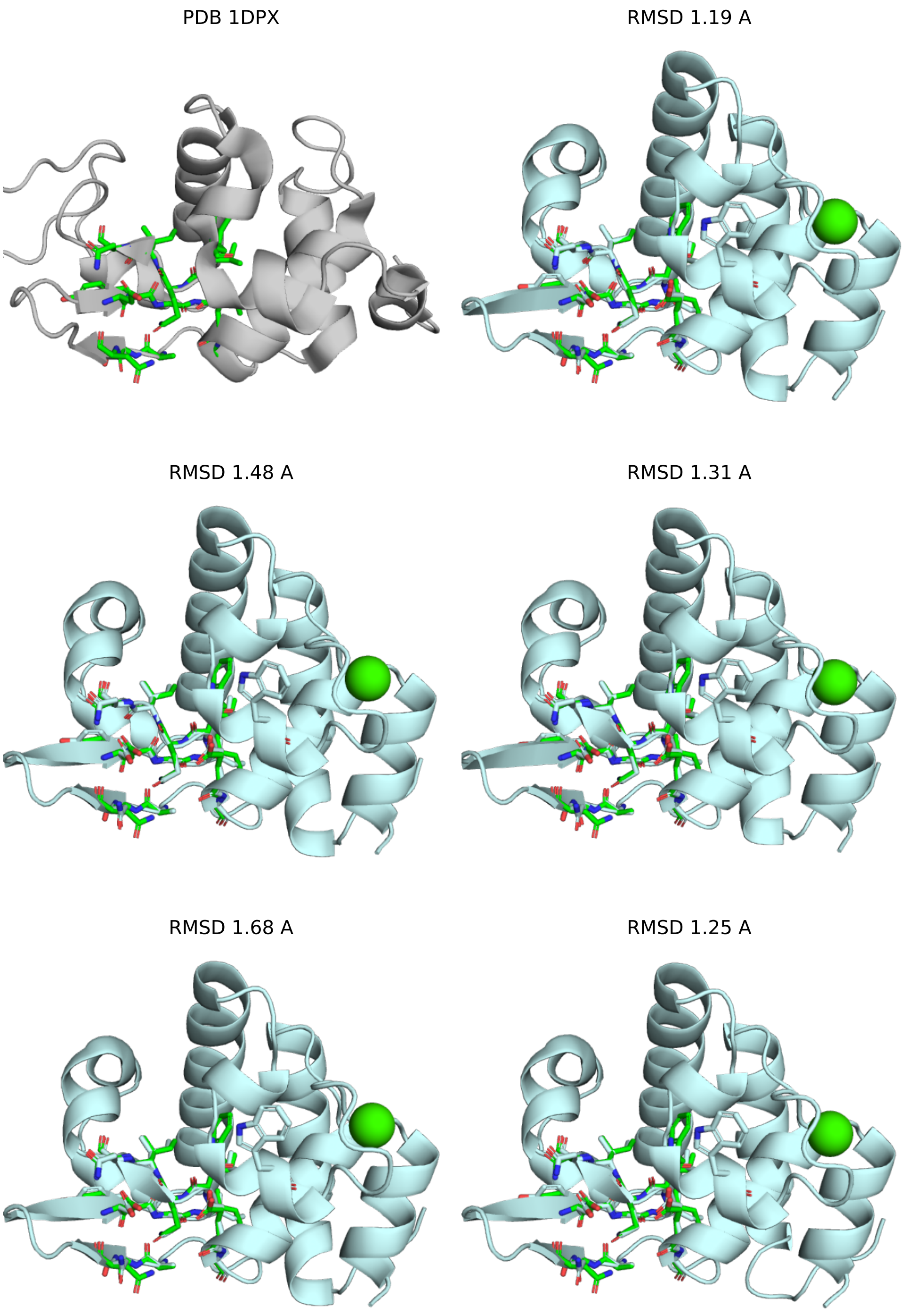

Figure S12: **Bound states of the lysozyme motif scaffold**. See previous caption.

bioRxiv preprint doi: https://doi.org/10.1101/2025.09.03.672144; this version posted September 4, 2025. The copyright holder for this preprint (which was not certified by peer review) is the author/funder, who has granted bioRxiv a license to display the preprint in perpetuity. It is made available under aCC-BY 4.0 International license.

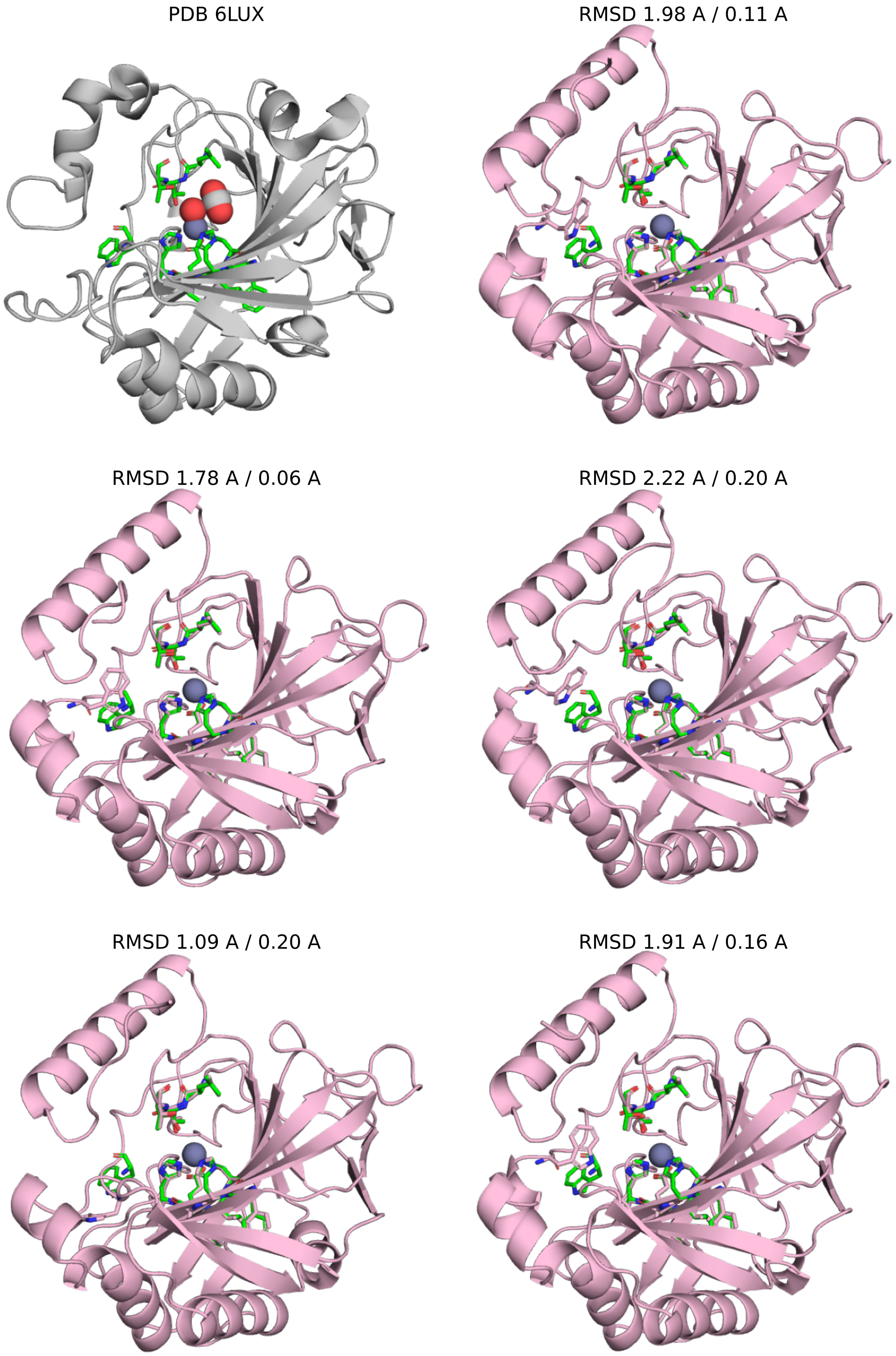

Figure S13: **Unbound states of the carbonic anhydrase motif scaffold**. Active site motif of carbonic anhydrase shown within PDB 6LUX (top left), with zinc cofactor and hydroxide and carbon dioxide substrates shown. The remaining structures show the five Chai-1 structure predictions without the calcium effector. The first RMSD listed is the Cα RMSD across all motif residues. These are somewhat larger than typical cutoff of 1 Å and we found it was dominated by the placement of a tryptophan residue (left) whose impact on catalytic activity was unclear. Thus, we filtered based on Cα RMSD of the four catalytic residues (second RMSD listed).

bioRxiv preprint doi: https://doi.org/10.1101/2025.09.03.672144; this version posted September 4, 2025. The copyright holder for this preprint (which was not certified by peer review) is the author/funder, who has granted bioRxiv a license to display the preprint in perpetuity. It is made available under aCC-BY 4.0 International license.

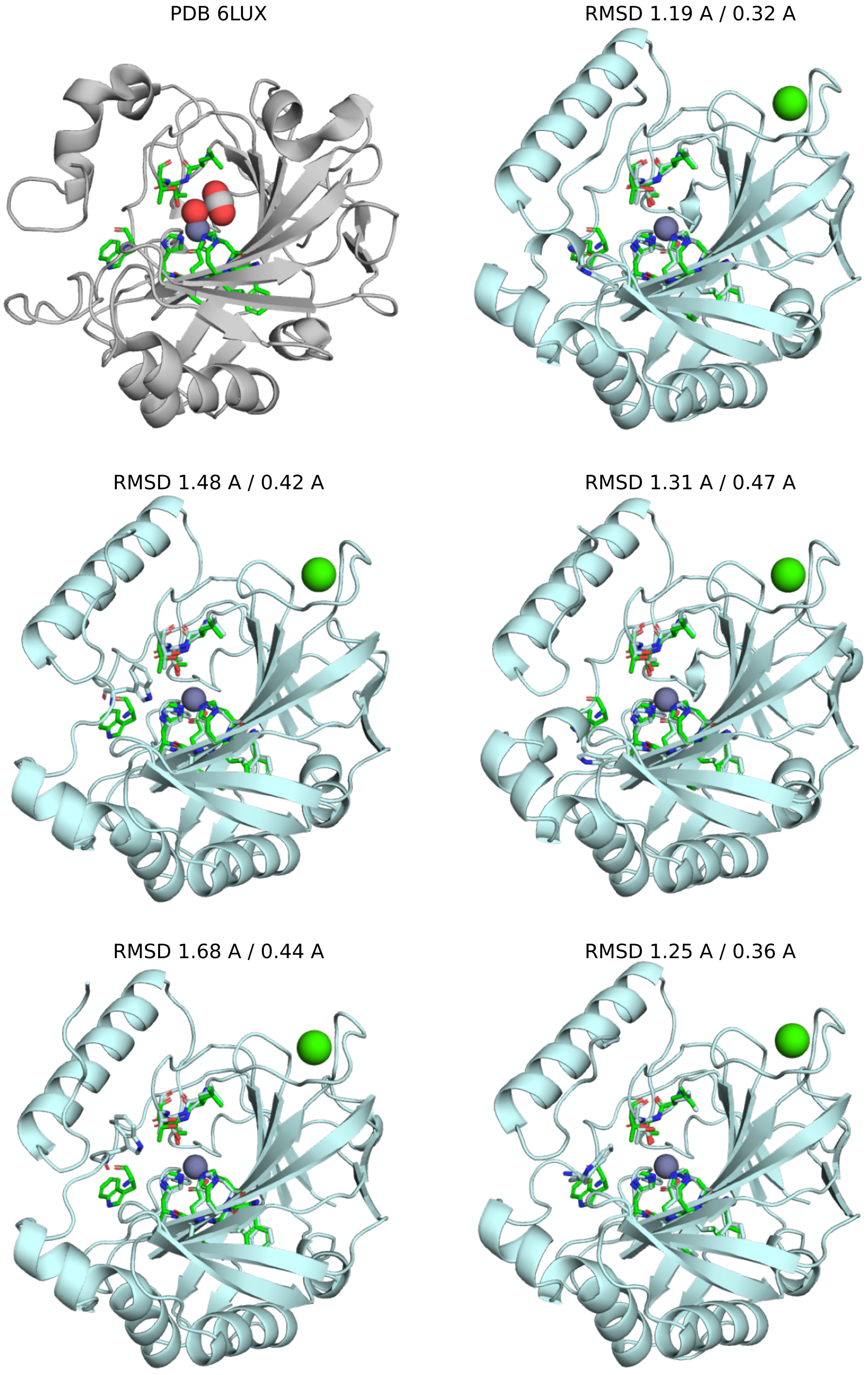

Figure S14: **Bound states of the carbonic anhydrase motif scaffold**. See previous caption.

bioRxiv preprint doi: https://doi.org/10.1101/2025.09.03.672144; this version posted September 4, 2025. The copyright holder for this preprint (which was not certified by peer review) is the author/funder, who has granted bioRxiv a license to display the preprint in perpetuity. It is made available under aCC-BY 4.0 International license.

Table S1: Descriptions and results for all 915 evaluated molecular function GO terms.

Term	Description	Occurrences	Successes	scTM	TM-diversity	TM-novelty (min)
GO:0017076	purine nucleotide binding	22956413	97	0.93	0.63	
GO:0097367	carbohydrate derivative binding	22382726	96	0.92	0.48	
GO:0032553	ribonucleotide binding	21512585	98	0.92	0.56	
GO:0032555	purine ribonucleotide binding	20786702	97	0.92	0.52	
GO:0030554	adenyl nucleotide binding	20626611	98	0.92	0.50	
GO:0035639	purine ribonucleoside triphosphate binding	20495784	99	0.92	0.49	
GO:0032559	adenyl ribonucleotide binding	18459894	100	0.92	0.52	
GO:0005524	ATP binding	18273940	97	0.92	0.48	
GO:0005215	transporter activity	17136396	98	0.93	0.52	
GO:0003677	DNA binding	16963614	67	0.85	0.57	
GO:0022857	transmembrane transporter activity	16898359	93	0.91	0.56	
GO:0140096	catalytic activity, acting on a protein	14398009	63	0.89	0.60	
GO:0016772	transferase activity, transferring phosphorus-containing groups	12146957	93	0.95	0.68	
GO:0003723	RNA binding	9077378	12	0.86	0.29	
GO:0016817	hydrolase activity, acting on acid anhydrides	8859070	64	0.88	0.49	
GO:0016818	hydrolase activity, acting on acid anhydrides, in phosphorus-containing anhydrid...	8811641	63	0.90	0.53	
GO:0016462	pyrophosphatase activity	8727052	45	0.88	0.52	
GO:0016788	hydrolase activity, acting on ester bonds	8569359	10	0.89	0.49	
GO:0046914	transition metal ion binding	7929417	6	0.76	0.45	
GO:0017111	nucleoside-triphosphatase activity	7827489	60	0.87	0.50	
GO:0140110	transcription regulator activity	7808154	66	0.67	0.37	
GO:0022804	active transmembrane transporter activity	7645552	39	0.89	0.42	
GO:0016301	kinase activity	7580968	91	0.95	0.69	
GO:0015075	ion transmembrane transporter activity	7337937	35	0.79	0.39	
GO:0015318	inorganic molecular entity transmembrane transporter activity	7243753	7	0.79	0.34	
GO:0003700	DNA-binding transcription factor activity	7091981	69	0.70	0.38	
GO:0008324	cation transmembrane transporter activity	6718093	5	0.81	0.38	
GO:0022890	inorganic cation transmembrane transporter activity	6404986	2	0.76	0.25	
GO:0008233	peptidase activity	6259124	32	0.86	0.43	
GO:0016887	ATPase activity	6242595	52	0.87	0.53	
GO:0140098	catalytic activity, acting on RNA	6210175	11	0.73	0.35	
GO:0016829	lyase activity	6098165	14	0.89	0.63	
GO:0016853	isomerase activity	6066403	2	0.93	0.82	
GO:0016773	phosphotransferase activity, alcohol group as acceptor	5777744	100	0.95	0.81	
GO:0015399	primary active transmembrane transporter activity	5615335	21	0.89	0.57	
GO:0016874	ligase activity	5509331	23	0.90	0.50	
GO:0043565	sequence-specific DNA binding	5029292	7	0.76	0.43	
GO:0140097	catalytic activity, acting on DNA	5011076	12	0.85	0.38	
GO:0005198	structural molecule activity	4966114	35	0.66	0.31	
GO:0016746	transferase activity, transferring acyl groups	4894433	65	0.84	0.66	
GO:0009055	electron transfer activity	4656696	13	0.56	0.36	
GO:0015078	proton transmembrane transporter activity	4585439	2	0.59	0.32	
GO:0008270	zinc ion binding	4544060	1	0.80		
GO:0098772	molecular function regulator	4433343	2	0.79	0.60	
GO:0016741	transferase activity, transferring one-carbon groups	4286583	99	0.91	0.65	
GO:0004672	protein kinase activity	3988854	97	0.93	0.78	
GO:0022853	active ion transmembrane transporter activity	3958268	0	0.83		
GO:0004518	nuclease activity	3954313	33	0.83	0.46	
GO:0046906	tetrapyrrole binding	3931047	33	0.67	0.38	
GO:0016779	nucleotidyltransferase activity	3901657	32	0.86	0.41	
GO:0003690	double-stranded DNA binding	3804794	1	0.88		
GO:0008168	methyltransferase activity	3800185	99	0.92	0.66	
GO:0051540	metal cluster binding	3667718	96	0.94	0.73	
GO:0051536	iron-sulfur cluster binding	3666092	98	0.95	0.73	
GO:0016757	transferase activity, transferring glycosyl groups	3572759	17	0.93	0.55	
GO:0020037	heme binding	3537432	34	0.65	0.41	
GO:1990837	sequence-specific double-stranded DNA binding	3373045	3	0.76	0.36	
GO:0003735	structural constituent of ribosome	3325740	37	0.50	0.26	
GO:0016747	transferase activity, transferring acyl groups other than amino-acyl groups	3319577	72	0.84	0.66	
GO:0004175	endopeptidase activity	3263101	6	0.89	0.54	
GO:0001067	regulatory region nucleic acid binding	3225086	0	0.77		
GO:0000976	transcription regulatory region sequence-specific DNA binding	3224990	2	0.78	0.59	
GO:0000287	magnesium ion binding	3103773	6	0.92	0.51	
GO:0016810	hydrolase activity, acting on carbon-nitrogen (but not peptide) bonds	2994620	12	0.86	0.59	
GO:0016614	oxidoreductase activity, acting on CH-OH group of donors	2922896	20	0.89	0.48	
GO:0042578	phosphoric ester hydrolase activity	2756712	10	0.88	0.49	
GO:0140101	catalytic activity, acting on a tRNA	2724981	36	0.80	0.42	
GO:0016798	hydrolase activity, acting on glycosyl bonds	2679691	14	0.87	0.53	
GO:0019842	vitamin binding	2651082	83	0.85	0.65	
GO:0060089	molecular transducer activity	2646736	92	0.94	0.83	
GO:0004519	endonuclease activity	2573275	15	0.77	0.38	
GO:0016616	oxidoreductase activity, acting on the CH-OH group of donors, NAD or NADP as acc...	2569384	69	0.93	0.65	
GO:0019001	guanyl nucleotide binding	2476666	94	0.82	0.56	
GO:0032561	guanyl ribonucleotide binding	2474301	86	0.83	0.57	
GO:0050660	flavin adenine dinucleotide binding	2388837	36	0.89	0.43	
GO:0005525	GTP binding	2359411	86	0.83	0.57	0.71 (0.56)
GO:0022803	passive transmembrane transporter activity	2300712	37	0.77	0.67	
GO:0015267	channel activity	2300711	42	0.79	0.61	
GO:0042626	ATPase-coupled transmembrane transporter activity	2295752	22	0.89	0.58	
GO:0016835	carbon-oxygen lyase activity	2229362	5	0.90	0.50	
GO:0030234	enzyme regulator activity	2103061	4	0.89	0.49	
GO:0016879	ligase activity, forming carbon-nitrogen bonds	2095688	23	0.91	0.55	
GO:0019843	rRNA binding	2078018	18	0.53	0.25	
GO:0051539	4 iron, 4 sulfur cluster binding	2074241	95	0.93	0.72	
GO:0004553	hydrolase activity, hydrolyzing O-glycosyl compounds	2051554	15	0.88	0.57	
GO:0016830	carbon-carbon lyase activity	2051524	32	0.96	0.70	
GO:0016791	phosphatase activity	2048037	49	0.88	0.76	
GO:0016675	oxidoreductase activity, acting on a heme group of donors	2042281	0	0.89		
GO:0008757	S-adenosylmethionine-dependent methyltransferase activity	2032461	15	0.92	0.67	
GO:0004129	cytochrome-c oxidase activity	2029875	1	0.84		
GO:0008237	metallopeptidase activity	2014105	10	0.87	0.49	
GO:0046873	metal ion transmembrane transporter activity	1922269	0	0.84		
GO:0015291	secondary active transmembrane transporter activity	1908900	45	0.89	0.40	0.84 (0.44)
GO:0044877	protein-containing complex binding	1890539	0	0.90		
GO:0017171	serine hydrolase activity	1870912	65	0.91	0.63	0.89 (0.77)
GO:0008236	serine-type peptidase activity	1852427	61	0.91	0.71	0.90 (0.77)
GO:0005506	iron ion binding	1849327	51	0.89	0.67	0.91 (0.86)
GO:0005216	ion channel activity	1815831	2	0.72	0.26	
GO:0038023	signaling receptor activity	1811055	93	0.92	0.82	0.89 (0.59)
GO:0004386	helicase activity	1783635	69	0.88	0.59	0.83 (0.62)
GO:0016836	hydro-lyase activity	1732784	0	0.80		
GO:0000981	DNA-binding transcription factor activity, RNA polymerase II-specific	1725696	4	0.84	0.38	

bioRxiv preprint doi: https://doi.org/10.1101/2025.09.03.672144; this version posted September 4, 2025. The copyright holder for this preprint (which was not certified by peer review) is the author/funder, who has granted bioRxiv a license to display the preprint in perpetuity. It is made available under aCC-BY 4.0 International license.

Term	Description	Occurrences	Successes	scTM	TM-diversity	TM-novelty (min)
GO:0140299	small molecule sensor activity	1718536	52	0.74	0.61	0.82 (0.60)
GO:0070279	vitamin B6 binding	1696410	84	0.83	0.66	0.81 (0.61)
GO:0030170	pyridoxal phosphate binding	1696403	87	0.84	0.64	0.81 (0.60)
GO:0016705	oxidoreductase activity, acting on paired donors, with incorporation or reductio...	1673658	50	0.92	0.66	0.91 (0.80)
GO:0004497	monooxygenase activity	1672575	73	0.93	0.64	0.91 (0.80)
GO:0016651	oxidoreductase activity, acting on NAD(P)H	1671443	22	0.91	0.81	0.91 (0.83)
GO:0004674	protein serine/threonine kinase activity	1649792	97	0.95	0.80	0.88 (0.74)
GO:0008094	DNA-dependent ATPase activity	1634241	3	0.88	0.79	
GO:0016758	transferase activity, transferring hexosyl groups	1614240	17	0.91	0.69	0.88 (0.73)
GO:0016775	phosphotransferase activity, nitrogenous group as acceptor	1566284	53	0.73	0.59	0.81 (0.55)
GO:0003924	GTPase activity	1553656	33	0.83	0.55	0.69 (0.58)
GO:0008238	exopeptidase activity	1546233	5	0.89	0.52	
GO:0016765	transferase activity, transferring alkyl or aryl (other than methyl) groups	1511695	1	0.96		
GO:0004673	protein histidine kinase activity	1506423	56	0.74	0.62	0.83 (0.62)
GO:0016627	oxidoreductase activity, acting on the CH-CH group of donors	1453300	1	0.85		
GO:0000155	phosphorelay sensor kinase activity	1443870	49	0.76	0.63	0.82 (0.62)
GO:0004888	transmembrane signaling receptor activity	1433020	88	0.92	0.81	0.89 (0.53)
GO:0000977	RNA polymerase II transcription regulatory region sequence-specific DNA binding	1427564	7	0.74	0.65	
GO:0008092	cytoskeletal protein binding	1400761	4	0.85	0.58	
GO:0051287	NAD binding	1387693	68	0.94	0.64	0.90 (0.75)
GO:0008514	organic anion transmembrane transporter activity	1349918	0	0.92		
GO:0140359	ABC-type transporter activity	1347792	1	0.89		
GO:0016875	ligase activity, forming carbon-oxygen bonds	1343584	42	0.81	0.44	0.80 (0.46)
GO:0004812	aminoacyl-tRNA ligase activity	1343583	34	0.78	0.46	0.81 (0.49)
GO:0004540	ribonuclease activity	1338388	12	0.67	0.46	0.64 (0.51)
GO:0016811	hydrolase activity, acting on carbon-nitrogen (but not peptide) bonds, in linear...	1306946	9	0.78	0.37	
GO:0005509	calcium ion binding	1274794	3	0.79	0.32	
GO:0019899	enzyme binding	1270249	0	0.90		
GO:0016769	transferase activity, transferring nitrogenous groups	1261805	56	0.86	0.70	0.84 (0.67)
GO:0046983	protein dimerization activity	1256161	0	0.82		
GO:0000049	tRNA binding	1255859	0	0.80		
GO:0016655	oxidoreductase activity, acting on NAD(P)H, quinone or similar compound as accep...	1254605	3	0.81	0.48	
GO:0030246	carbohydrate binding	1246103	0	0.80		
GO:0008289	lipid binding	1246092	2	0.76	0.47	
GO:0000987	cis-regulatory region sequence-specific DNA binding	1241391	0	0.78		
GO:0008483	transaminase activity	1233439	40	0.86	0.69	0.83 (0.72)
GO:0016831	carboxy-lyase activity	1227333	1	0.96		
GO:0004527	exonuclease activity	1225169	13	0.83	0.46	0.68 (0.64)
GO:0000978	RNA polymerase II cis-regulatory region sequence-specific DNA binding	1212767	1	0.75		
GO:0004252	serine-type endopeptidase activity	1203621	52	0.89	0.71	0.90 (0.77)
GO:0005261	cation channel activity	1201026	5	0.71	0.31	
GO:0005342	organic acid transmembrane transporter activity	1197798	0	0.94		
GO:0046943	carboxylic acid transmembrane transporter activity	1194014	0	0.92		
GO:0003729	mRNA binding	1191978	1	0.84		
GO:0034061	DNA polymerase activity	1160534	1	0.84		
GO:0016903	oxidoreductase activity, acting on the aldehyde or oxo group of donors	1158157	26	0.89	0.51	0.87 (0.49)
GO:0060090	molecular adaptor activity	1144566	0	0.90		
GO:0052689	carboxylic ester hydrolase activity	1117280	75	0.92	0.75	0.83 (0.72)
GO:0050661	NADP binding	1108426	28	0.93	0.62	0.87 (0.64)
GO:0003954	NADH dehydrogenase activity	1068866	0	0.90		
GO:0008173	RNA methyltransferase activity	1051776	46	0.82	0.66	0.76 (0.53)
GO:0005102	signaling receptor binding	1047779	2	0.66	0.44	
GO:0004222	metalloendopeptidase activity	1039567	2	0.90	0.47	
GO:0030674	protein-macromolecule adaptor activity	1032013	0	0.83		
GO:0016755	transferase activity, transferring amino-acyl groups	992216	0	0.74		
GO:0016209	antioxidant activity	984696	8	0.66	0.58	
GO:0016407	acetyltransferase activity	978586	73	0.82	0.56	0.68 (0.49)
GO:1901681	sulfur compound binding	962044	16	0.85	0.53	0.83 (0.53)
GO:0016763	transferase activity, transferring pentosyl groups	960603	0	0.88		
GO:0097747	RNA polymerase activity	935811	1	0.54		
GO:0034062	5'-3' RNA polymerase activity	935806	0	0.49		
GO:0016866	intramolecular transferase activity	930429	14	0.81	0.56	0.71 (0.58)
GO:0043021	ribonucleoprotein complex binding	925662	0	0.79		
GO:0015103	inorganic anion transmembrane transporter activity	902290	1	0.81		
GO:0051213	dioxygenase activity	894093	23	0.91	0.82	0.89 (0.70)
GO:0016667	oxidoreductase activity, acting on a sulfur group of donors	881281	43	0.60	0.43	0.54 (0.37)
GO:0008194	UDP-glycosyltransferase activity	877645	32	0.89	0.74	0.87 (0.81)
GO:0004536	deoxyribonuclease activity	862098	15	0.88	0.73	0.77 (0.70)
GO:0004521	endoribonuclease activity	854063	9	0.67	0.48	
GO:0019787	ubiquitin-like protein transferase activity	849661	5	0.69	0.31	
GO:0016620	oxidoreductase activity, acting on the aldehyde or oxo group of donors, NAD or N...	847303	51	0.90	0.62	0.91 (0.57)
GO:0003678	DNA helicase activity	837294	13	0.88	0.54	0.84 (0.73)
GO:0016782	transferase activity, transferring sulfur-containing groups	836827	2	0.93	0.85	
GO:0008137	NADH dehydrogenase (ubiquinone) activity	836817	0	0.83		
GO:0060589	nucleoside-triphosphatase regulator activity	828247	4	0.88	0.67	
GO:0030695	GTPase regulator activity	828247	6	0.88	0.41	
GO:0051537	2 iron, 2 sulfur cluster binding	821490	9	0.72	0.43	
GO:0022836	gated channel activity	816718	4	0.67	0.31	
GO:0043022	ribosome binding	813766	0	0.72		
GO:0051082	unfolded protein binding	798764	16	0.69	0.36	0.62 (0.46)
GO:0015293	symporter activity	797934	20	0.91	0.68	0.86 (0.73)
GO:0008170	N-methyltransferase activity	797048	62	0.85	0.52	0.76 (0.46)
GO:0004842	ubiquitin-protein transferase activity	792861	5	0.72	0.30	
GO:0071949	FAD binding	789552	31	0.93	0.68	0.86 (0.75)
GO:0015297	antiporter activity	784942	26	0.91	0.57	0.87 (0.75)
GO:0016854	racemase and epimerase activity	781790	6	0.90	0.62	
GO:0016298	lipase activity	756077	1	0.90		
GO:0004930	G protein-coupled receptor activity	739118	92	0.94	0.84	0.91 (0.75)
GO:0010181	FMN binding	725734	30	0.75	0.60	0.82 (0.53)
GO:0008047	enzyme activator activity	723085	1	0.85		
GO:0048038	quinone binding	710241	0	0.66		
GO:0003779	actin binding	702220	1	0.86		
GO:0004180	carboxypeptidase activity	697021	3	0.87	0.74	
GO:0008408	3'-5' exonuclease activity	695714	5	0.78	0.54	
GO:0016410	N-acyltransferase activity	685071	84	0.79	0.57	0.69 (0.52)
GO:0016881	acid-amino acid ligase activity	670208	10	0.95	0.69	
GO:0016860	intramolecular oxidoreductase activity	670193	0	0.89		
GO:0019829	ATPase-coupled cation transmembrane transporter activity	663252	28	0.76	0.52	0.79 (0.53)
GO:0004721	phosphoprotein phosphatase activity	659947	91	0.89	0.78	0.88 (0.56)
GO:0016987	sigma factor activity	655629	79	0.57	0.43	0.54 (0.38)
GO:0043177	organic acid binding	655428	4	0.83	0.60	
GO:0016684	oxidoreductase activity, acting on peroxide as acceptor	642951	23	0.79	0.65	0.84 (0.49)
GO:0031406	carboxylic acid binding	641396	3	0.87	0.55	
GO:0016877	ligase activity, forming carbon-sulfur bonds	640204	28	0.91	0.67	0.89 (0.73)
GO:0070001	aspartic-type peptidase activity	639411	28	0.92	0.75	0.89 (0.72)
GO:0004190	aspartic-type endopeptidase activity	639366	31	0.92	0.76	0.89 (0.75)

bioRxiv preprint doi: https://doi.org/10.1101/2025.09.03.672144; this version posted September 4, 2025. The copyright holder for this preprint (which was not certified by peer review) is the author/funder, who has granted bioRxiv a license to display the preprint in perpetuity. It is made available under aCC-BY 4.0 International license.

Term	Description	Occurrences	Successes	scTM	TM-diversity	TM-novelty (min)
GO:0003712	transcription coregulator activity	637013	0	0.76		
GO:0005543	phospholipid binding	626473	1	0.60		
GO:0004601	peroxidase activity	623942	27	0.84	0.81	0.89 (0.78)
GO:0033218	amide binding	622759	2	0.86	0.28	
GO:0015081	sodium ion transmembrane transporter activity	620734	0	0.81		
GO:0005319	lipid transporter activity	619574	1	0.79		
GO:0000156	phosphorelay response regulator activity	618575	11	0.58	0.48	0.67 (0.50)
GO:0008234	cysteine-type peptidase activity	611038	26	0.88	0.73	0.86 (0.75)
GO:0016814	hydrolase activity, acting on carbon-nitrogen (but not peptide) bonds, in cyclic...	601834	13	0.85	0.40	0.53 (0.44)
GO:0042802	identical protein binding	599947	0	0.89		
GO:0015252	proton channel activity	597603	7	0.70	0.62	
GO:0015144	carbohydrate transmembrane transporter activity	597099	5	0.88	0.65	
GO:0003746	translation elongation factor activity	594921	7	0.76	0.54	
GO:0061659	ubiquitin-like protein ligase activity	593105	1	0.71		
GO:0140102	catalytic activity, acting on a rRNA	591267	2	0.83	0.60	
GO:0044183	protein folding chaperone	590515	6	0.74	0.60	
GO:0003887	DNA-directed DNA polymerase activity	581719	7	0.80	0.43	
GO:0008649	rRNA methyltransferase activity	581636	2	0.81	0.63	
GO:0003899	DNA-directed 5'-3' RNA polymerase activity	580828	0	0.46		
GO:0046933	proton-transporting ATP synthase activity, rotational mechanism	577013	8	0.66	0.68	
GO:0022884	macromolecule transmembrane transporter activity	573967	46	0.68	0.56	0.67 (0.46)
GO:0005507	copper ion binding	569354	57	0.80	0.67	0.81 (0.55)
GO:0015631	tubulin binding	567778	2	0.88	0.67	
GO:0046915	transition metal ion transmembrane transporter activity	564447	0	0.82		
GO:0016884	carbon-nitrogen ligase activity, with glutamine as amido-N-donor	562214	47	0.82	0.58	0.76 (0.56)
GO:0004177	aminopeptidase activity	560516	9	0.90	0.53	
GO:0004857	enzyme inhibitor activity	559708	13	0.67	0.40	0.73 (0.44)
GO:0061630	ubiquitin protein ligase activity	559277	1	0.76		
GO:0016628	oxidoreductase activity, acting on the CH-CH group of donors, NAD or NADP as acc...	555290	0	0.95		
GO:0008235	metalloexopeptidase activity	553331	0	0.94		
GO:0015079	potassium ion transmembrane transporter activity	545752	0	0.81		
GO:0008081	phosphoric diester hydrolase activity	544643	1	0.89		
GO:0016645	oxidoreductase activity, acting on the CH-NH group of donors	535656	1	0.87		
GO:0008080	N-acetyltransferase activity	530315	95	0.74	0.53	0.67 (0.53)
GO:0016780	phosphotransferase activity, for other substituted phosphate groups	529932	4	0.79	0.70	
GO:0008276	protein methyltransferase activity	527611	4	0.81	0.58	
GO:0016776	phosphotransferase activity, phosphate group as acceptor	521507	11	0.90	0.59	0.64 (0.60)
GO:0015036	disulfide oxidoreductase activity	511010	28	0.66	0.41	0.53 (0.40)
GO:0003682	chromatin binding	502923	0	0.90		
GO:0004520	endodeoxyribonuclease activity	500131	5	0.81	0.71	
GO:0016891	endoribonuclease activity, producing 5'-phosphomonoesters	490474	6	0.65	0.55	
GO:0016799	hydrolase activity, hydrolyzing N-glycosyl compounds	490295	0	0.91		
GO:0003697	single-stranded DNA binding	489432	0	0.78		
GO:0016859	cis-trans isomerase activity	486912	70	0.64	0.35	0.62 (0.43)
GO:0030145	manganese ion binding	486286	2	0.82	0.69	
GO:0008017	microtubule binding	486051	9	0.90	0.87	
GO:0008374	O-acyltransferase activity	482662	0	0.88		
GO:0016878	acid-thiol ligase activity	480335	31	0.90	0.74	0.91 (0.66)
GO:0016840	carbon-nitrogen lyase activity	479167	1	0.76		
GO:0016861	intramolecular oxidoreductase activity, interconverting aldoses and ketoses	476753	3	0.89	0.52	
GO:0070566	adenylyltransferase activity	470900	3	0.86	0.58	
GO:0016783	sulfurtransferase activity	462118	1	0.82		
GO:0016679	oxidoreductase activity, acting on diphenols and related substances as donors	461632	0	0.79		
GO:0051015	actin filament binding	459208	0	0.76		
GO:0019200	carbohydrate kinase activity	457830	32	0.95	0.82	0.90 (0.79)
GO:0042910	xenobiotic transmembrane transporter activity	455111	5	0.82	0.78	
GO:0003684	damaged DNA binding	454263	5	0.75	0.77	
GO:0019239	deaminase activity	452147	4	0.54	0.49	
GO:0003755	peptidyl-prolyl cis-trans isomerase activity	442470	65	0.66	0.37	0.64 (0.48)
GO:0042277	peptide binding	440930	0	0.80		
GO:0016638	oxidoreductase activity, acting on the CH-NH2 group of donors	437700	0	0.74		
GO:0003724	RNA helicase activity	432940	51	0.88	0.71	0.89 (0.64)
GO:0019205	nucleobase-containing compound kinase activity	428538	45	0.69	0.43	0.63 (0.45)
GO:0003743	translation initiation factor activity	427712	1	0.89		
GO:0005085	guanyl-nucleotide exchange factor activity	422714	1	0.85		
GO:0061783	peptidoglycan muralytic activity	412307	7	0.69	0.38	
GO:0003774	motor activity	411820	30	0.73	0.69	0.88 (0.45)
GO:0008509	anion transmembrane transporter activity	408132	10	0.80	0.54	
GO:0016857	racemase and epimerase activity, acting on carbohydrates and derivatives	406365	2	0.95	0.93	
GO:0015562	efflux transmembrane transporter activity	401819	0	0.72		
GO:0005096	GTPase activator activity	398956	0	0.87		
GO:0030545	receptor regulator activity	398720	0	0.50		
GO:0140104	molecular carrier activity	396509	2	0.83	0.47	
GO:0018455	alcohol dehydrogenase [NAD(P)+] activity	386197	0	0.94		
GO:0042393	histone binding	384803	0	0.93		
GO:0016405	CoA-ligase activity	383092	14	0.93	0.66	0.90 (0.82)
GO:0030546	signaling receptor activator activity	382539	0	0.50		
GO:0022834	ligand-gated channel activity	380244	9	0.68	0.43	
GO:0015276	ligand-gated ion channel activity	380221	5	0.72	0.49	
GO:0048018	receptor ligand activity	379124	0	0.52		
GO:0070008	serine-type exopeptidase activity	375821	21	0.86	0.65	0.85 (0.73)
GO:0140318	protein transporter activity	375362	56	0.70	0.56	0.68 (0.54)
GO:0015035	protein disulfide oxidoreductase activity	373674	23	0.64	0.40	0.52 (0.41)
GO:0015926	glucosidase activity	373288	12	0.88	0.67	0.88 (0.78)
GO:0042887	amide transmembrane transporter activity	372597	0	0.77		
GO:0035091	phosphatidylinositol binding	372390	0	0.55		
GO:0016701	oxidoreductase activity, acting on single donors with incorporation of molecular...	371853	0	0.75		
GO:0016646	oxidoreductase activity, acting on the CH-NH group of donors, NAD or NADP as acc...	371626	0	0.64		
GO:0008320	protein transmembrane transporter activity	369556	41	0.70	0.56	0.67 (0.46)
GO:0003995	acyl-CoA dehydrogenase activity	368669	1	0.84		
GO:0004620	phospholipase activity	366350	1	0.85		
GO:0030976	thiamine pyrophosphate binding	366135	70	0.80	0.65	0.83 (0.56)
GO:0002161	aminoacyl-tRNA editing activity	363767	0	0.70		
GO:0004659	prenyltransferase activity	363634	34	0.96	0.85	0.91 (0.86)
GO:0019207	kinase regulator activity	362615	2	0.84	0.67	
GO:0016812	hydrolase activity, acting on carbon-nitrogen (but not peptide) bonds, in cyclic...	362310	0	0.88		
GO:0016790	thiolester hydrolase activity	360404	1	0.91		
GO:0016832	aldehyde-lyase activity	358997	17	0.95	0.68	0.71 (0.62)
GO:0008171	O-methyltransferase activity	358278	5	0.79	0.62	
GO:0004532	exoribonuclease activity	358046	3	0.86	0.37	
GO:0016896	exoribonuclease activity, producing 5'-phosphomonoesters	356272	2	0.84	0.67	
GO:0051087	chaperone binding	355704	0	0.65		
GO:0003968	RNA-directed 5'-3' RNA polymerase activity	355397	0	0.63		
GO:0008175	tRNA methyltransferase activity	354939	0	0.83		
GO:0033293	monocarboxylic acid binding	354050	7	0.88	0.81	
GO:0022832	voltage-gated channel activity	353268	1	0.78		

bioRxiv preprint doi: https://doi.org/10.1101/2025.09.03.672144; this version posted September 4, 2025. The copyright holder for this preprint (which was not certified by peer review) is the author/funder, who has granted bioRxiv a license to display the preprint in perpetuity. It is made available under aCC-BY 4.0 International license.

Term	Description	Occurrences	Successes	scTM	TM-diversity	TM-novelty (min)
GO:0005244	voltage-gated ion channel activity	348850	1	0.79		
GO:0009982	pseudouridine synthase activity	347828	12	0.80	0.59	0.72 (0.64)
GO:0016849	phosphorus-oxygen lyase activity	347026	2	0.83	0.71	
GO:0043138	3'-5' DNA helicase activity	345757	1	0.84		
GO:1901505	carbohydrate derivative transmembrane transporter activity	344573	0	0.94		
GO:0019213	deacetylase activity	344273	38	0.93	0.80	0.91 (0.82)
GO:0050839	cell adhesion molecule binding	343855	0	0.65		
GO:0019900	kinase binding	341591	0	0.86		
GO:0016725	oxidoreductase activity, acting on CH or CH2 groups	338182	0	0.81		
GO:0019887	protein kinase regulator activity	336984	4	0.86	0.74	
GO:0008121	ubiquinol-cytochrome-c reductase activity	335075	0	0.64		
GO:0004713	protein tyrosine kinase activity	334566	5	0.93	0.84	
GO:0019104	DNA N-glycosylase activity	334066	2	0.79	0.60	
GO:0004529	exodeoxyribonuclease activity	332639	0	0.90		
GO:0003916	DNA topoisomerase activity	332125	0	0.58		
GO:0022829	wide pore channel activity	329047	83	0.78	0.66	0.73 (0.64)
GO:0015085	calcium ion transmembrane transporter activity	327655	0	0.73		
GO:0004185	serine-type carboxypeptidase activity	325789	11	0.85	0.61	0.84 (0.78)
GO:0016833	oxo-acid-lyase activity	321911	4	0.92	0.68	
GO:0016744	transferase activity, transferring aldehyde or ketonic groups	320870	2	0.82	0.66	
GO:0016742	hydroxymethyl-, formyl- and related transferase activity	312014	52	0.78	0.66	0.73 (0.63)
GO:0016668	oxidoreductase activity, acting on a sulfur group of donors, NAD(P) as acceptor	308942	13	0.82	0.79	0.90 (0.86)
GO:0019901	protein kinase binding	306556	0	0.86		
GO:0016885	ligase activity, forming carbon-carbon bonds	304114	21	0.76	0.63	0.71 (0.50)
GO:0016846	carbon-sulfur lyase activity	299307	0	0.55		
GO:0016706	2-oxoglutarate-dependent dioxygenase activity	298609	25	0.93	0.83	0.90 (0.66)
GO:0016895	exodeoxyribonuclease activity, producing 5'-phosphomonoesters	296545	0	0.89		
GO:0015288	porin activity	294099	82	0.77	0.67	0.74 (0.65)
GO:0042625	ATPase-coupled ion transmembrane transporter activity	293538	17	0.73	0.49	0.39 (0.35)
GO:0044769	ATPase activity, coupled to transmembrane movement of ions, rotational mechanism	293538	0	0.69		
GO:0046961	proton-transporting ATPase activity, rotational mechanism	293534	0	0.68		
GO:1904680	peptide transmembrane transporter activity	289117	0	0.92		
GO:0015108	chloride transmembrane transporter activity	288037	0	0.82		
GO:0070006	metalloaminopeptidase activity	285995	1	0.85		
GO:0043531	ADP binding	284214	0	0.79		
GO:0016709	oxidoreductase activity, acting on paired donors, with incorporation or reductio...	282920	14	0.85	0.48	0.89 (0.81)
GO:0016421	CoA carboxylase activity	278143	21	0.76	0.66	0.75 (0.68)
GO:0061134	peptidase regulator activity	277655	13	0.73	0.37	0.69 (0.45)
GO:0015343	siderophore transmembrane transporter activity	277010	0	0.80		
GO:0004176	ATP-dependent peptidase activity	276609	0	0.79		
GO:0016868	intramolecular transferase activity, phosphotransferases	276230	7	0.91	0.45	
GO:0072341	modified amino acid binding	272661	1	0.86		
GO:0016702	oxidoreductase activity, acting on single donors with incorporation of molecular...	269657	0	0.73		
GO:0016774	phosphotransferase activity, carboxyl group as acceptor	269512	8	0.94	0.75	
GO:0016841	ammonia-lyase activity	268283	0	0.75		
GO:0019238	cyclohydrolase activity	267098	0	0.72		
GO:0047429	nucleoside-triphosphate diphosphatase activity	264998	1	0.67		
GO:0004197	cysteine-type endopeptidase activity	264740	3	0.88	0.82	
GO:1901682	sulfur compound transmembrane transporter activity	264310	1	0.85		
GO:0016743	carboxyl- or carbamoyltransferase activity	264082	0	0.92		
GO:0015344	siderophore uptake transmembrane transporter activity	263969	0	0.81		
GO:0003714	transcription corepressor activity	262340	0	0.78		
GO:0009975	cyclase activity	260126	0	0.82		
GO:0051920	peroxiredoxin activity	259891	21	0.60	0.41	0.57 (0.49)
GO:0016597	amino acid binding	254807	0	0.93		
GO:0031419	cobalamin binding	254366	2	0.84	0.49	
GO:0004523	RNA-DNA hybrid ribonuclease activity	254340	1	0.67		
GO:0005253	anion channel activity	254123	0	0.79		
GO:0004315	3-oxoacyl-[acyl-carrier-protein] synthase activity	250204	6	0.93	0.63	
GO:0046982	protein heterodimerization activity	249572	41	0.48	0.45	0.58 (0.35)
GO:0022843	voltage-gated cation channel activity	248850	0	0.67		
GO:0070403	NAD+ binding	248521	0	0.85		
GO:0048029	monosaccharide binding	244902	5	0.84	0.66	
GO:0030414	peptidase inhibitor activity	244760	25	0.68	0.36	0.67 (0.45)
GO:0005516	calmodulin binding	244194	0	0.89		
GO:0004725	protein tyrosine phosphatase activity	243683	85	0.90	0.81	0.90 (0.59)
GO:0004722	protein serine/threonine phosphatase activity	242119	47	0.90	0.80	0.87 (0.78)
GO:0016408	C-acyltransferase activity	241558	33	0.84	0.75	0.91 (0.79)
GO:0000175	3'-5'-exoribonuclease activity	232878	2	0.86	0.74	
GO:0008658	penicillin binding	232739	5	0.86	0.66	
GO:0005048	signal sequence binding	229591	0	0.95		
GO:0005267	potassium channel activity	227662	4	0.68	0.42	
GO:0061135	endopeptidase regulator activity	226608	21	0.73	0.42	0.73 (0.41)
GO:0051020	GTPase binding	226175	0	0.88		
GO:0101005	ubiquitinyl hydrolase activity	226115	41	0.92	0.87	0.92 (0.83)
GO:0016289	CoA hydrolase activity	225380	0	0.88		
GO:0004030	aldehyde dehydrogenase [NAD(P)+] activity	222506	42	0.92	0.81	0.95 (0.90)
GO:0140030	modification-dependent protein binding	220696	0	0.84		
GO:0046912	transferase activity, transferring acyl groups, acyl groups converted into alkyl...	219651	0	0.79		
GO:0031267	small GTPase binding	219031	0	0.90		
GO:0003918	DNA topoisomerase type II (double strand cut, ATP-hydrolyzing) activity	218942	0	0.59		
GO:0016855	racemase and epimerase activity, acting on amino acids and derivatives	216524	4	0.91	0.64	
GO:0004866	endopeptidase inhibitor activity	216430	17	0.70	0.35	0.64 (0.43)
GO:0004022	alcohol dehydrogenase (NAD+) activity	216214	0	0.93		
GO:0008409	5'-3' exonuclease activity	216117	0	0.90		
GO:0000149	SNARE binding	212890	0	0.79		
GO:0001664	G protein-coupled receptor binding	212007	0	0.72		
GO:0004843	thiol-dependent ubiquitin-specific protease activity	211934	53	0.92	0.87	0.92 (0.86)
GO:0008422	beta-glucosidase activity	210397	12	0.87	0.62	0.89 (0.81)
GO:0003727	single-stranded RNA binding	210015	2	0.74	0.49	
GO:0005539	glycosaminoglycan binding	209916	0	0.43		
GO:0019904	protein domain specific binding	206750	0	0.70		
GO:0004029	aldehyde dehydrogenase (NAD+) activity	206208	11	0.93	0.71	0.96 (0.92)
GO:0016984	ribulose-bisphosphate carboxylase activity	205698	0	0.91		
GO:0050145	nucleoside monophosphate kinase activity	205183	24	0.73	0.54	0.67 (0.56)
GO:0042803	protein homodimerization activity	205135	0	0.82		
GO:0016880	acid-ammonia (or amide) ligase activity	204864	1	0.89		
GO:0099094	ligand-gated cation channel activity	204815	0	0.63		
GO:0005262	calcium channel activity	204609	0	0.68		
GO:0031177	phosphopantetheine binding	204490	0	0.85		
GO:0070567	cytidylyltransferase activity	200864	2	0.74	0.73	
GO:0034212	peptide N-acetyltransferase activity	199824	0	0.68		
GO:0008172	S-methyltransferase activity	198088	0	0.60		
GO:0003713	transcription coactivator activity	197855	0	0.74		
GO:0005254	chloride channel activity	197536	0	0.77		
GO:0016838	carbon-oxygen lyase activity, acting on phosphates	196873	0	0.95		

bioRxiv preprint doi: https://doi.org/10.1101/2025.09.03.672144; this version posted September 4, 2025. The copyright holder for this preprint (which was not certified by peer review) is the author/funder, who has granted bioRxiv a license to display the preprint in perpetuity. It is made available under aCC-BY 4.0 International license.

Term	Description	Occurrences	Successes	scTM	TM-diversity	TM-novelty (min)
GO:0016837	carbon-oxygen lyase activity, acting on polysaccharides	196843	1	0.84		
GO:0047661	amino-acid racemase activity	196575	7	0.90	0.69	
GO:0004806	triglyceride lipase activity	196099	0	0.94		
GO:0008198	ferrous iron binding	195695	0	0.82		
GO:0016778	diphosphotransferase activity	195419	5	0.68	0.52	
GO:1901981	phosphatidylinositol phosphate binding	194666	0	0.74		
GO:0043178	alcohol binding	193651	0	0.82		
GO:0009008	DNA-methyltransferase activity	192797	10	0.84	0.64	
GO:0019208	phosphatase regulator activity	192040	0	0.91		
GO:0016151	nickel cation binding	188640	0	0.82		
GO:0004359	glutaminase activity	188116	5	0.88	0.79	
GO:0046527	glucosyltransferase activity	187437	6	0.89	0.71	
GO:0003725	double-stranded RNA binding	186664	0	0.77		
GO:0046812	host cell surface binding	186003	0	0.63		
GO:0046789	host cell surface receptor binding	185633	0	0.84		
GO:0004033	aldo-keto reductase (NADP) activity	184654	28	0.94	0.86	0.92 (0.79)
GO:0032182	ubiquitin-like protein binding	183324	0	0.78		
GO:0070181	small ribosomal subunit rRNA binding	181132	0	0.48		
GO:0016888	endodeoxyribonuclease activity, producing 5'-phosphomonoesters	180109	4	0.94	0.81	
GO:0004364	glutathione transferase activity	180045	18	0.83	0.61	0.67 (0.56)
GO:0016722	oxidoreductase activity, oxidizing metal ions	179506	0	0.78		
GO:0043546	molybdopterin cofactor binding	178603	0	0.73		
GO:0019888	protein phosphatase regulator activity	178589	0	0.90		
GO:0008146	sulfotransferase activity	178506	3	0.91	0.82	
GO:0019202	amino acid kinase activity	178328	0	0.97		
GO:0005249	voltage-gated potassium channel activity	177385	0	0.65		
GO:0043023	ribosomal large subunit binding	174957	0	0.78		
GO:0001653	peptide receptor activity	174954	5	0.94	0.86	
GO:0016813	hydrolase activity, acting on carbon-nitrogen (but not peptide) bonds, in linear...	174814	2	0.90	0.71	
GO:0003777	microtubule motor activity	173936	56	0.83	0.75	0.92 (0.53)
GO:0016417	S-acyltransferase activity	172012	0	0.80		
GO:0016413	O-acetyltransferase activity	170516	2	0.86	0.79	
GO:0070402	NADPH binding	169977	1	0.97		
GO:0008134	transcription factor binding	168192	0	0.81		
GO:0008484	sulfuric ester hydrolase activity	168079	1	0.82		
GO:0043130	ubiquitin binding	167722	0	0.80		
GO:0070180	large ribosomal subunit rRNA binding	166128	7	0.59	0.28	
GO:0050897	cobalt ion binding	164184	0	0.80		
GO:0051119	sugar transmembrane transporter activity	164029	0	0.69		
GO:0019203	carbohydrate phosphatase activity	163095	0	0.93		
GO:0003906	DNA-(apurinic or apyrimidinic site) endonuclease activity	162862	0	0.87		
GO:0008252	nucleotidase activity	162804	0	0.92		
GO:0003988	acetyl-CoA C-acyltransferase activity	161603	38	0.86	0.76	0.91 (0.79)
GO:0003989	acetyl-CoA carboxylase activity	161496	6	0.77	0.66	
GO:0008982	protein-N(PI)-phosphohistidine-sugar phosphotransferase activity	161158	0	0.83		
GO:0008079	translation termination factor activity	160891	11	0.55	0.39	0.54 (0.38)
GO:0003747	translation release factor activity	160834	9	0.52	0.42	
GO:0070569	uridylyltransferase activity	160831	1	0.87		
GO:0030594	neurotransmitter receptor activity	160718	11	0.78	0.75	0.88 (0.81)
GO:0050308	sugar-phosphatase activity	160589	0	0.91		
GO:0005125	cytokine activity	159637	0	0.50		
GO:0016886	ligase activity, forming phosphoric ester bonds	159546	21	0.81	0.69	0.82 (0.76)
GO:0047134	protein-disulfide reductase activity	159034	0	0.83		
GO:0003730	mRNA 3'-UTR binding	157053	0	0.83		
GO:0016435	rRNA (guanine) methyltransferase activity	156873	2	0.76	0.61	
GO:0047617	acyl-CoA hydrolase activity	155267	0	0.92		
GO:0031072	heat shock protein binding	152679	28	0.62	0.40	0.58 (0.36)
GO:0016671	oxidoreductase activity, acting on a sulfur group of donors, disulfide as accept...	151809	0	0.64		
GO:0035251	UDP-glucosyltransferase activity	151466	1	0.90		
GO:0030151	molybdenum ion binding	151176	0	0.70		
GO:0015925	galactosidase activity	151121	0	0.86		
GO:0001882	nucleoside binding	149828	0	0.39		
GO:0016712	oxidoreductase activity, acting on paired donors, with incorporation or reductio...	149272	0	0.93		
GO:0016728	oxidoreductase activity, acting on CH or CH2 groups, disulfide as acceptor	149030	0	0.83		
GO:0032549	ribonucleoside binding	148548	1	0.38		
GO:0005548	phospholipid transporter activity	147516	0	0.71		
GO:0019199	transmembrane receptor protein kinase activity	147284	12	0.95	0.83	0.91 (0.89)
GO:0070063	RNA polymerase binding	146461	0	0.71		
GO:0001216	DNA-binding transcription activator activity	144929	0	0.69		
GO:0016842	amidine-lyase activity	144759	5	0.78	0.62	
GO:0009002	serine-type D-Ala-D-Ala carboxypeptidase activity	144645	0	0.84		
GO:0016805	dipeptidase activity	144534	0	0.88		
GO:0004748	ribonucleoside-diphosphate reductase activity, thioredoxin disulfide as acceptor	143802	0	0.83		
GO:0061731	ribonucleoside-diphosphate reductase activity	143802	0	0.83		
GO:0008199	ferric iron binding	143626	0	0.74		
GO:0016661	oxidoreductase activity, acting on other nitrogenous compounds as donors	142766	0	0.74		
GO:0008528	G protein-coupled peptide receptor activity	141652	0	0.94		
GO:0005200	structural constituent of cytoskeleton	141217	41	0.82	0.75	0.90 (0.75)
GO:0015145	monosaccharide transmembrane transporter activity	140757	20	0.92	0.69	0.88 (0.78)
GO:0044389	ubiquitin-like protein ligase binding	139168	0	0.75		
GO:0016615	malate dehydrogenase activity	138933	19	0.90	0.85	0.89 (0.78)
GO:0016278	lysine N-methyltransferase activity	138489	0	0.84		
GO:0016279	protein-lysine N-methyltransferase activity	138487	0	0.84		
GO:0070568	guanylyltransferase activity	137554	8	0.92	0.79	
GO:0004112	cyclic-nucleotide phosphodiesterase activity	137408	12	0.92	0.85	0.94 (0.87)
GO:0030551	cyclic nucleotide binding	136113	0	0.51		
GO:0016453	C-acetyltransferase activity	136095	15	0.86	0.82	0.91 (0.79)
GO:0004300	enoyl-CoA hydratase activity	136028	19	0.86	0.74	0.81 (0.69)
GO:0016168	chlorophyll binding	135298	0	0.41		
GO:0005230	extracellular ligand-gated ion channel activity	134950	23	0.81	0.79	0.90 (0.80)
GO:0016433	rRNA (adenine) methyltransferase activity	134300	0	0.83		
GO:0000150	recombinase activity	133802	0	0.66		
GO:0043024	ribosomal small subunit binding	133464	0	0.77		
GO:0008745	N-acetylmuramoyl-L-alanine amidase activity	132782	0	0.69		
GO:0004457	lactate dehydrogenase activity	132604	17	0.92	0.75	0.89 (0.61)
GO:0016641	oxidoreductase activity, acting on the CH-NH2 group of donors, oxygen as accepto...	132222	0	0.53		
GO:0042054	histone methyltransferase activity	131874	67	0.60	0.54	0.65 (0.54)
GO:0060590	ATPase regulator activity	131859	0	0.75		
GO:0003796	lysozyme activity	131568	1	0.70		
GO:0016882	cyclo-ligase activity	131477	0	0.85		
GO:0004867	serine-type endopeptidase inhibitor activity	131355	21	0.68	0.31	0.54 (0.43)
GO:0004596	peptide alpha-N-acetyltransferase activity	130998	0	0.63		
GO:0003909	DNA ligase activity	130798	16	0.79	0.66	0.81 (0.72)
GO:0005126	cytokine receptor binding	130687	0	0.53		
GO:0004549	tRNA-specific ribonuclease activity	130472	0	0.88		
GO:0004550	nucleoside diphosphate kinase activity	129618	54	0.59	0.52	0.66 (0.52)

bioRxiv preprint doi: https://doi.org/10.1101/2025.09.03.672144; this version posted September 4, 2025. The copyright holder for this preprint (which was not certified by peer review) is the author/funder, who has granted bioRxiv a license to display the preprint in perpetuity. It is made available under aCC-BY 4.0 International license.

Term	Description	Occurrences	Successes	scTM	TM-diversity	TM-novelty (min)
GO:0004396	hexokinase activity	129154	5	0.91	0.65	
GO:0008379	thioredoxin peroxidase activity	128653	1	0.65		
GO:0004040	amidase activity	128580	0	0.66		
GO:0008083	growth factor activity	128255	0	0.54		
GO:0031625	ubiquitin protein ligase binding	127226	0	0.68		
GO:0016211	ammonia ligase activity	127146	0	0.88		
GO:0004792	thiosulfate sulfurtransferase activity	126733	3	0.90	0.61	
GO:0019209	kinase activator activity	126228	0	0.94		
GO:0016423	tRNA (guanine) methyltransferase activity	126219	0	0.77		
GO:0030295	protein kinase activator activity	125086	0	0.89		
GO:0050497	transferase activity, transferring alkylthio groups	124481	46	0.89	0.62	0.82 (0.52)
GO:0008641	ubiquitin-like modifier activating enzyme activity	124419	77	0.82	0.71	0.79 (0.68)
GO:0016997	alpha-sialidase activity	123365	0	0.93		
GO:0017116	single-stranded DNA helicase activity	123289	0	0.81		
GO:0004308	exo-alpha-sialidase activity	123268	0	0.89		
GO:0070492	oligosaccharide binding	122506	0	0.90		
GO:0008296	3'-5'-exodeoxyribonuclease activity	122442	0	0.89		
GO:0016634	oxidoreductase activity, acting on the CH-CH group of donors, oxygen as acceptor	122047	0	0.73		
GO:0005496	steroid binding	121906	0	0.62		
GO:0030247	polysaccharide binding	121859	0	0.56		
GO:0070003	threonine-type peptidase activity	121768	4	0.67	0.53	
GO:0016208	AMP binding	120190	0	0.81		
GO:0051117	ATPase binding	119932	0	0.67		
GO:0004791	thioredoxin-disulfide reductase activity	119540	1	0.76		
GO:0008061	chitin binding	119224	31	0.59	0.65	0.87 (0.68)
GO:0008106	alcohol dehydrogenase (NADP+) activity	119030	14	0.95	0.87	0.92 (0.82)
GO:0008410	CoA-transferase activity	118984	0	0.79		
GO:0008395	steroid hydroxylase activity	118669	0	0.93		
GO:0008253	5'-nucleotidase activity	118026	0	0.91		
GO:0032451	demethylase activity	117409	0	0.84		
GO:1902936	phosphatidylinositol bisphosphate binding	116902	0	0.85		
GO:0004714	transmembrane receptor protein tyrosine kinase activity	115999	0	0.93		
GO:0015923	mannosidase activity	115861	0	0.79		
GO:0016682	oxidoreductase activity, acting on diphenols and related substances as donors, o...	115149	0	0.65		
GO:0016229	steroid dehydrogenase activity	114186	0	0.91		
GO:0016639	oxidoreductase activity, acting on the CH-NH2 group of donors, NAD or NADP as ac...	113639	7	0.86	0.58	
GO:0015450	P-P-bond-hydrolysis-driven protein transmembrane transporter activity	113279	83	0.69	0.60	0.70 (0.48)
GO:0003688	DNA replication origin binding	113128	0	0.82		
GO:0045156	electron transporter, transferring electrons within the cyclic electron transpor...	112773	0	0.42		
GO:0045296	cadherin binding	112694	0	0.73		
GO:0019210	kinase inhibitor activity	111643	0	0.75		
GO:0051538	3 iron, 4 sulfur cluster binding	111245	2	0.74	0.37	
GO:0004312	fatty acid synthase activity	111193	18	0.82	0.64	0.89 (0.75)
GO:0008443	phosphofructokinase activity	110770	28	0.86	0.73	0.87 (0.79)
GO:0004860	protein kinase inhibitor activity	109362	0	0.72		
GO:0016247	channel regulator activity	109309	0	0.88		
GO:0042562	hormone binding	109244	0	0.66		
GO:0004000	adenosine deaminase activity	109130	0	0.97		
GO:0031420	alkali metal ion binding	107342	0	0.76		
GO:0016538	cyclin-dependent protein serine/threonine kinase regulator activity	107306	2	0.84	0.77	
GO:0004764	shikimate 3-dehydrogenase (NADP+) activity	107144	2	0.94	0.80	
GO:0004731	purine-nucleoside phosphorylase activity	106641	2	0.91	0.76	
GO:0031490	chromatin DNA binding	106252	0	0.82		
GO:0098960	postsynaptic neurotransmitter receptor activity	106177	5	0.77	0.79	
GO:0016863	intramolecular oxidoreductase activity, transposing C=C bonds	105994	0	0.85		
GO:0048030	disaccharide binding	105877	0	0.90		
GO:0004181	metallocarboxypeptidase activity	105742	2	0.93	0.86	
GO:0008242	omega peptidase activity	105199	0	0.66		
GO:0022824	transmitter-gated ion channel activity	105050	4	0.77	0.80	
GO:0022835	transmitter-gated channel activity	105050	6	0.76	0.75	
GO:0008239	dipeptidyl-peptidase activity	104787	0	0.78		
GO:0032934	sterol binding	103880	0	0.61		
GO:0099106	ion channel regulator activity	103681	0	0.88		
GO:0140223	general transcription initiation factor activity	103467	0	0.87		
GO:0009041	uridylate kinase activity	103186	0	0.84		
GO:0008840	4-hydroxy-tetrahydrodipicolinate synthase activity	101408	1	0.93		
GO:0003823	antigen binding	101339	0	0.67		
GO:0140375	immune receptor activity	101229	1	0.52		
GO:0106130	purine phosphoribosyltransferase activity	101220	38	0.61	0.51	0.53 (0.45)
GO:1904047	S-adenosyl-L-methionine binding	100025	11	0.91	0.67	0.75 (0.63)
GO:0052745	inositol phosphate phosphatase activity	99479	50	0.95	0.79	0.83 (0.76)
GO:0005549	odorant binding	99450	0	0.84		
GO:0030276	clathrin binding	99167	0	0.87		
GO:0090729	toxin activity	99166	1	0.79		
GO:0050136	NADH dehydrogenase (quinone) activity	98827	0	0.81		
GO:0003855	3-dehydroquinate dehydratase activity	96857	0	0.84		
GO:0016822	hydrolase activity, acting on acid carbon-carbon bonds	96299	0	0.73		
GO:0016823	hydrolase activity, acting on acid carbon-carbon bonds, in ketonic substances	96297	0	0.75		
GO:0005504	fatty acid binding	96242	0	0.77		
GO:0030291	protein serine/threonine kinase inhibitor activity	95388	0	0.67		
GO:0004322	ferroxidase activity	95039	0	0.83		
GO:0016724	oxidoreductase activity, oxidizing metal ions, oxygen as acceptor	95039	0	0.82		
GO:0008097	5S rRNA binding	94984	0	0.48		
GO:0140297	DNA-binding transcription factor binding	94976	0	0.92		
GO:0098531	ligand-activated transcription factor activity	94954	1	0.77		
GO:0004879	nuclear receptor activity	94890	2	0.79	0.68	
GO:0043891	glyceraldehyde-3-phosphate dehydrogenase (NAD(P)+) (phosphorylating) activity	94775	33	0.89	0.80	0.90 (0.85)
GO:0004834	tryptophan synthase activity	94526	1	0.93		
GO:0009881	photoreceptor activity	94518	1	0.95		
GO:0004365	glyceraldehyde-3-phosphate dehydrogenase (NAD+) (phosphorylating) activity	94301	28	0.90	0.81	0.90 (0.85)
GO:0030955	potassium ion binding	93971	0	0.76		
GO:1901982	maltose binding	92877	0	0.90		
GO:0031491	nucleosome binding	92324	0	0.94		
GO:0000104	succinate dehydrogenase activity	92085	0	0.70		
GO:0016748	succinyltransferase activity	91428	0	0.85		
GO:0004089	carbonate dehydratase activity	91332	1	0.73		
GO:0140034	methylation-dependent protein binding	90791	0	0.73		
GO:0019212	phosphatase inhibitor activity	90433	0	0.52		
GO:0004896	cytokine receptor activity	90357	0	0.53		
GO:0042834	peptidoglycan binding	90349	0	0.42		
GO:0016801	hydrolase activity, acting on ether bonds	90047	0	0.94		
GO:0035064	methylated histone binding	89875	0	0.70		
GO:0016803	ether hydrolase activity	89708	0	0.95		
GO:0005179	hormone activity	89038	0	0.58		
GO:0120013	lipid transfer activity	89032	0	0.80		
GO:0005178	integrin binding	88855	0	0.73		

bioRxiv preprint doi: https://doi.org/10.1101/2025.09.03.672144; this version posted September 4, 2025. The copyright holder for this preprint (which was not certified by peer review) is the author/funder, who has granted bioRxiv a license to display the preprint in perpetuity. It is made available under aCC-BY 4.0 International license.

Term	Description	Occurrences	Successes	scTM	TM-diversity	TM-novelty (min)
GO:0016251	RNA polymerase II general transcription initiation factor activity	88449	0	0.88		
GO:0019825	oxygen binding	88439	11	0.67	0.54	0.66 (0.54)
GO:0004565	beta-galactosidase activity	88416	0	0.81		
GO:0035438	cyclic-di-GMP binding	88299	0	0.51		
GO:0004590	orotidine-5'-phosphate decarboxylase activity	87823	0	0.92		
GO:0003955	NAD(P)H dehydrogenase (quinone) activity	87744	0	0.86		
GO:0016635	oxidoreductase activity, acting on the CH-CH group of donors, quinone or related...	86780	0	0.66		
GO:0019905	syntaxin binding	86767	0	0.83		
GO:0016730	oxidoreductase activity, acting on iron-sulfur proteins as donors	86672	0	0.81		
GO:0004124	cysteine synthase activity	85914	8	0.94	0.89	
GO:0004427	inorganic diphosphatase activity	84917	0	0.65		
GO:0003993	acid phosphatase activity	84881	1	0.88		
GO:0004017	adenylate kinase activity	84862	21	0.78	0.61	0.68 (0.62)
GO:0004864	protein phosphatase inhibitor activity	84732	0	0.49		
GO:0016160	amylase activity	84411	1	0.91		
GO:0004148	dihydrolipoyl dehydrogenase activity	84293	0	0.86		
GO:0005544	calcium-dependent phospholipid binding	84017	1	0.81		
GO:0106310	protein serine kinase activity	83197	0	0.92		
GO:0015929	hexosaminidase activity	83067	0	0.86		
GO:0004568	chitinase activity	82641	13	0.90	0.81	0.92 (0.87)
GO:0099529	neurotransmitter receptor activity involved in regulation of postsynaptic membra...	82302	0	0.78		
GO:1904315	transmitter-gated ion channel activity involved in regulation of postsynaptic me...	82225	2	0.78	0.85	
GO:0019955	cytokine binding	81949	0	0.53		
GO:0004045	aminoacyl-tRNA hydrolase activity	81568	2	0.66	0.56	
GO:0097506	deaminated base DNA N-glycosylase activity	81342	0	0.78		
GO:0004844	uracil DNA N-glycosylase activity	81340	0	0.78		
GO:0016426	tRNA (adenine) methyltransferase activity	81261	0	0.43		
GO:0004448	isocitrate dehydrogenase activity	80443	0	0.83		
GO:0120227	acyl-CoA binding	79466	0	0.84		
GO:0004556	alpha-amylase activity	78847	0	0.90		
GO:0016703	oxidoreductase activity, acting on single donors with incorporation of molecular...	78560	0	0.82		
GO:0019206	nucleoside kinase activity	78259	1	0.85		
GO:0001217	DNA-binding transcription repressor activity	78251	0	0.73		
GO:0140142	nucleocytoplasmic carrier activity	78120	0	0.94		
GO:0140078	class I DNA-(apurinic or apyrimidinic site) endonuclease activity	77966	0	0.83		
GO:0004645	1,4-alpha-oligoglucan phosphorylase activity	77815	0	0.87		
GO:0030515	snoRNA binding	77321	0	0.85		
GO:0004114	3',5'-cyclic-nucleotide phosphodiesterase activity	77176	12	0.92	0.88	0.95 (0.93)
GO:0016721	oxidoreductase activity, acting on superoxide radicals as acceptor	76863	4	0.66	0.52	
GO:0033764	steroid dehydrogenase activity, acting on the CH-OH group of donors, NAD or NADP...	76840	0	0.94		
GO:1901567	fatty acid derivative binding	76815	0	0.89		
GO:0000062	fatty-acyl-CoA binding	76785	0	0.85		
GO:0004640	phosphoribosylanthranilate isomerase activity	76311	0	0.75		
GO:0019902	phosphatase binding	75964	0	0.88		
GO:0032296	double-stranded RNA-specific ribonuclease activity	75608	4	0.70	0.51	
GO:0004525	ribonuclease III activity	75603	3	0.70	0.60	
GO:0042162	telomeric DNA binding	75557	0	0.73		
GO:0043175	RNA polymerase core enzyme binding	75471	0	0.76		
GO:0061733	peptide-lysine-N-acetyltransferase activity	75073	0	0.78		
GO:0008201	heparin binding	75051	0	0.56		
GO:0017056	structural constituent of nuclear pore	74472	0	0.79		
GO:0004459	L-lactate dehydrogenase activity	74414	38	0.95	0.89	0.94 (0.86)
GO:0005546	phosphatidylinositol-4,5-bisphosphate binding	74214	0	0.81		
GO:0004623	phospholipase A2 activity	74203	0	0.68		
GO:0004784	superoxide dismutase activity	74175	3	0.69	0.58	
GO:0008800	beta-lactamase activity	74086	0	0.85		
GO:0019838	growth factor binding	73901	0	0.90		
GO:0008897	holo-[acyl-carrier-protein] synthase activity	73451	0	0.86		
GO:0015038	glutathione disulfide oxidoreductase activity	73388	0	0.63		
GO:0140414	phosphopantetheine-dependent carrier activity	72951	1	0.74		
GO:0004106	chorismate mutase activity	72673	0	0.48		
GO:0004332	fructose-bisphosphate aldolase activity	72616	0	0.96		
GO:0004831	tyrosine-tRNA ligase activity	72406	28	0.92	0.75	0.84 (0.77)
GO:0051192	prosthetic group binding	71238	1	0.74		
GO:0044620	ACP phosphopantetheine attachment site binding	71237	0	0.78		
GO:0000036	acyl carrier activity	71237	0	0.80		
GO:0008312	7S RNA binding	71012	0	0.77		
GO:0004096	catalase activity	70609	0	0.36		
GO:0044325	ion channel binding	70512	0	0.89		
GO:0015485	cholesterol binding	69630	0	0.62		
GO:0070401	NADP+ binding	69023	0	0.94		
GO:0004402	histone acetyltransferase activity	68538	0	0.84		
GO:0005324	long-chain fatty acid transporter activity	68175	0	0.80		
GO:0035591	signaling adaptor activity	68129	0	0.85		
GO:0004634	phosphopyruvate hydratase activity	68112	1	0.85		
GO:0005245	voltage-gated calcium channel activity	67966	0	0.64		
GO:0017069	snRNA binding	67622	0	0.91		
GO:0004526	ribonuclease P activity	66470	3	0.56	0.36	
GO:0042586	peptide deformylase activity	66451	1	0.65		
GO:0004347	glucose-6-phosphate isomerase activity	66366	3	0.81	0.70	
GO:0036402	proteasome-activating ATPase activity	66266	2	0.81	0.69	
GO:0004619	phosphoglycerate mutase activity	66265	0	0.78		
GO:0004372	glycine hydroxymethyltransferase activity	66253	0	0.83		
GO:0070851	growth factor receptor binding	66113	0	0.58		
GO:0001098	basal transcription machinery binding	66048	0	0.73		
GO:0001099	basal RNA polymerase II transcription machinery binding	66042	0	0.77		
GO:0000179	rRNA (adenine-N6,N6-)-dimethyltransferase activity	65590	1	0.90		
GO:0015666	restriction endodeoxyribonuclease activity	65512	0	0.73		
GO:0008716	D-alanine-D-alanine ligase activity	65500	0	0.96		
GO:0003910	DNA ligase (ATP) activity	65271	1	0.79		
GO:0061629	RNA polymerase II-specific DNA-binding transcription factor binding	65095	0	0.91		
GO:0031418	L-ascorbic acid binding	64743	0	0.79		
GO:0004316	3-oxoacyl-[acyl-carrier-protein] reductase (NADPH) activity	63867	35	0.89	0.81	0.83 (0.74)
GO:0140296	general transcription initiation factor binding	63469	4	0.65	0.42	
GO:0004563	beta-N-acetylhexosaminidase activity	62880	0	0.91		
GO:0008477	purine nucleosidase activity	62159	0	0.93		
GO:0004715	non-membrane spanning protein tyrosine kinase activity	62155	0	0.88		
GO:0017022	myosin binding	62079	0	0.66		
GO:0004807	triose-phosphate isomerase activity	61975	1	0.89		
GO:0005231	excitatory extracellular ligand-gated ion channel activity	61781	2	0.78	0.73	
GO:0016889	endodeoxyribonuclease activity, producing 3'-phosphomonoesters	61638	0	0.73		
GO:0019903	protein phosphatase binding	61183	0	0.91		
GO:0003857	3-hydroxyacyl-CoA dehydrogenase activity	61127	4	0.87	0.76	
GO:0003938	IMP dehydrogenase activity	60808	0	0.63		
GO:0008948	oxaloacetate decarboxylase activity	60562	0	0.74		
GO:0042083	5,10-methylenetetrahydrofolate-dependent methyltransferase activity	60447	0	0.81		

bioRxiv preprint doi: https://doi.org/10.1101/2025.09.03.672144; this version posted September 4, 2025. The copyright holder for this preprint (which was not certified by peer review) is the author/funder, who has granted bioRxiv a license to display the preprint in perpetuity. It is made available under aCC-BY 4.0 International license.

Term	Description	Occurrences	Successes	scTM	TM-diversity	TM-novelty (min)
GO:0004830	tryptophan-tRNA ligase activity	60297	2	0.93	0.82	
GO:0043539	protein serine/threonine kinase activator activity	59633	0	0.92		
GO:0008174	mRNA methyltransferase activity	59365	4	0.87	0.61	
GO:0045182	translation regulator activity	59127	0	0.79		
GO:0005344	oxygen carrier activity	58966	11	0.67	0.53	0.64 (0.59)
GO:0004799	thymidylate synthase activity	58788	0	0.80		
GO:0008066	glutamate receptor activity	57432	0	0.74		
GO:0004450	isocitrate dehydrogenase (NADP+) activity	57116	0	0.75		
GO:0003950	NAD+ ADP-ribosyltransferase activity	56999	0	0.70		
GO:0016273	arginine N-methyltransferase activity	56907	9	0.85	0.81	
GO:0016274	protein-arginine N-methyltransferase activity	56907	5	0.86	0.77	
GO:0004422	hypoxanthine phosphoribosyltransferase activity	56847	11	0.57	0.53	0.56 (0.52)
GO:0004618	phosphoglycerate kinase activity	56455	0	0.76		
GO:0004602	glutathione peroxidase activity	56445	0	0.69		
GO:0061650	ubiquitin-like protein conjugating enzyme activity	56308	36	0.66	0.52	0.59 (0.51)
GO:0004557	alpha-galactosidase activity	55838	0	0.91		
GO:0000993	RNA polymerase II complex binding	55653	0	0.76		
GO:0031369	translation initiation factor binding	55632	0	0.83		
GO:0022839	ion gated channel activity	55300	0	0.65		
GO:0016726	oxidoreductase activity, acting on CH or CH2 groups, NAD or NADP as acceptor	55254	0	0.75		
GO:0003952	NAD+ synthase (glutamine-hydrolyzing) activity	54234	12	0.77	0.63	0.68 (0.57)
GO:0036002	pre-mRNA binding	53968	0	0.52		
GO:0004585	ornithine carbamoyltransferase activity	53952	0	0.93		
GO:0030060	L-malate dehydrogenase activity	53907	50	0.91	0.87	0.91 (0.81)
GO:0008784	alanine racemase activity	53689	0	0.76		
GO:0004751	ribose-5-phosphate isomerase activity	53270	2	0.68	0.51	
GO:0004712	protein serine/threonine/tyrosine kinase activity	52858	0	0.94		
GO:0016670	oxidoreductase activity, acting on a sulfur group of donors, oxygen as acceptor	52569	0	0.48		
GO:0004298	threonine-type endopeptidase activity	52158	19	0.70	0.64	0.70 (0.63)
GO:0042132	fructose 1,6-bisphosphate 1-phosphatase activity	52113	17	0.95	0.79	0.81 (0.76)
GO:0004629	phospholipase C activity	52108	0	0.70		
GO:0016420	malonyltransferase activity	51857	50	0.94	0.89	0.93 (0.83)
GO:0016419	S-malonyltransferase activity	51809	54	0.94	0.88	0.93 (0.82)
GO:0004314	[acyl-carrier-protein] S-malonyltransferase activity	51809	48	0.92	0.89	0.93 (0.86)
GO:0008227	G protein-coupled amine receptor activity	50580	0	0.94		
GO:0016872	intramolecular lyase activity	50575	0	0.63		
GO:0042301	phosphate ion binding	50483	0	0.86		
GO:0046556	alpha-L-arabinofuranosidase activity	50450	0	0.89		
GO:0004156	dihydropteroate synthase activity	49975	2	0.95	0.72	
GO:0008839	4-hydroxy-tetrahydrodipicolinate reductase	49189	7	0.95	0.73	
GO:0061631	ubiquitin conjugating enzyme activity	48787	39	0.62	0.52	0.58 (0.51)
GO:0061608	nuclear import signal receptor activity	48698	0	0.96		
GO:0015616	DNA translocase activity	48403	0	0.87		
GO:0017025	TBP-class protein binding	47270	3	0.70	0.38	
GO:0008810	cellulase activity	46628	0	0.75		
GO:0005227	calcium activated cation channel activity	46100	0	0.67		
GO:0016631	enoyl-[acyl-carrier-protein] reductase activity	46043	0	0.91		
GO:0004340	glucokinase activity	46028	5	0.89	0.81	
GO:0000339	RNA cap binding	45753	0	0.84		
GO:0004146	dihydrofolate reductase activity	45388	1	0.70		
GO:0016731	oxidoreductase activity, acting on iron-sulfur proteins as donors, NAD or NADP a...	44648	0	0.89		
GO:0097599	xylanase activity	44080	0	0.87		
GO:0016695	oxidoreductase activity, acting on hydrogen as donor	43949	2	0.41	0.29	
GO:0005217	intracellular ligand-gated ion channel activity	43879	0	0.70		
GO:1990782	protein tyrosine kinase binding	43531	0	0.58		
GO:0004318	enoyl-[acyl-carrier-protein] reductase (NADH) activity	43314	0	0.91		
GO:0008795	NAD+ synthase activity	43133	4	0.81	0.61	
GO:0004170	dUTP diphosphatase activity	42889	14	0.43	0.59	0.69 (0.43)
GO:0038024	cargo receptor activity	42267	0	0.41		
GO:0008937	ferredoxin-NAD(P) reductase activity	42025	0	0.91		
GO:0004425	indole-3-glycerol-phosphate synthase activity	41761	1	0.78		
GO:0032813	tumor necrosis factor receptor superfamily binding	41738	0	0.50		
GO:0033818	beta-ketoacyl-acyl-carrier-protein synthase III activity	41686	10	0.96	0.83	
GO:0004514	nicotinate-nucleotide diphosphorylase (carboxylating) activity	41609	0	0.91		
GO:0001671	ATPase activator activity	41553	0	0.53		
GO:0004707	MAP kinase activity	41257	9	0.93	0.80	
GO:0051018	protein kinase A binding	40484	0	0.60		
GO:0004324	ferredoxin-NADP+ reductase activity	40483	0	0.92		
GO:0051219	phosphoprotein binding	39966	0	0.86		
GO:0003756	protein disulfide isomerase activity	39963	47	0.55	0.46	0.63 (0.51)
GO:0016864	intramolecular oxidoreductase activity, transposing S-S bonds	39963	54	0.53	0.46	0.62 (0.54)
GO:0016699	oxidoreductase activity, acting on hydrogen as donor, iron-sulfur protein as acc...	39010	0	0.44		
GO:0008901	ferredoxin hydrogenase activity	39010	0	0.45		
GO:0004567	beta-mannosidase activity	38746	0	0.85		
GO:0004482	mRNA (guanine-N7-)-methyltransferase activity	38510	2	0.84	0.46	
GO:0045309	protein phosphorylated amino acid binding	38374	0	0.64		
GO:0004069	L-aspartate:2-oxoglutarate aminotransferase activity	37777	0	0.77		
GO:0030971	receptor tyrosine kinase binding	37465	0	0.58		
GO:0001046	core promoter sequence-specific DNA binding	37260	0	0.67		
GO:0005536	glucose binding	37050	6	0.88	0.79	
GO:0031386	protein tag	36824	14	0.39	0.51	0.72 (0.54)
GO:0019003	GDP binding	36588	0	0.66		
GO:0019840	isoprenoid binding	36505	0	0.74		
GO:0004126	cytidine deaminase activity	36462	2	0.54	0.49	
GO:0031683	G-protein beta/gamma-subunit complex binding	36293	11	0.87	0.79	0.88 (0.82)
GO:0008138	protein tyrosine/serine/threonine phosphatase activity	36282	61	0.67	0.52	0.61 (0.55)
GO:0051879	Hsp90 protein binding	35773	0	0.89		
GO:0016917	GABA receptor activity	34681	0	0.84		
GO:0099604	ligand-gated calcium channel activity	34359	0	0.68		
GO:0016899	oxidoreductase activity, acting on the CH-OH group of donors, oxygen as acceptor	33613	0	0.89		
GO:0042800	histone methyltransferase activity (H3-K4 specific)	33562	0	0.58		
GO:0004970	ionotropic glutamate receptor activity	33271	0	0.67		
GO:0008013	beta-catenin binding	33221	0	0.67		
GO:0009678	pyrophosphate hydrolysis-driven proton transmembrane transporter activity	33198	0	0.74		
GO:0016504	peptidase activator activity	32834	0	0.90		
GO:0004032	alditol:NADP+ 1-oxidoreductase activity	32729	14	0.92	0.89	0.94 (0.91)
GO:0016892	endoribonuclease activity, producing 3'-phosphomonoesters	31944	0	0.57		
GO:0004551	nucleotide diphosphatase activity	31619	7	0.64	0.54	
GO:0016662	oxidoreductase activity, acting on other nitrogenous compounds as donors, cytoch...	30872	0	0.66		
GO:0042826	histone deacetylase binding	30860	0	0.78		
GO:0010333	terpene synthase activity	30523	4	0.87	0.71	
GO:0016843	amine-lyase activity	30019	0	0.86		
GO:0042379	chemokine receptor binding	29781	0	0.34		
GO:0002020	protease binding	29693	0	0.82		
GO:0050840	extracellular matrix binding	28977	0	0.49		
GO:0004675	transmembrane receptor protein serine/threonine kinase activity	28792	7	0.93	0.81	

bioRxiv preprint doi: https://doi.org/10.1101/2025.09.03.672144; this version posted September 4, 2025. The copyright holder for this preprint (which was not certified by peer review) is the author/funder, who has granted bioRxiv a license to display the preprint in perpetuity. It is made available under aCC-BY 4.0 International license.

Term	Description	Occurrences	Successes	scTM	TM-diversity	TM-novelty (min)
GO:0004869	cysteine-type endopeptidase inhibitor activity	28718	0	0.47		
GO:0052657	guanine phosphoribosyltransferase activity	28595	7	0.56	0.50	
GO:0008930	methylthioadenosine nucleosidase activity	28292	0	0.85		
GO:0008782	adenosylhomocysteine nucleosidase activity	27918	0	0.89		
GO:0001530	lipopolysaccharide binding	27747	0	0.63		
GO:0005044	scavenger receptor activity	27168	0	0.39		
GO:0030248	cellulose binding	26936	0	0.61		
GO:0048027	mRNA 5'-UTR binding	26521	0	0.49		
GO:0005518	collagen binding	25946	0	0.64		
GO:0004362	glutathione-disulfide reductase activity	25845	0	0.86		
GO:0015278	calcium-release channel activity	25745	0	0.68		
GO:2001070	starch binding	25373	0	0.67		
GO:0005212	structural constituent of eye lens	24863	0	0.45		
GO:0034511	U3 snoRNA binding	24693	0	0.81		
GO:0008009	chemokine activity	24518	0	0.37		
GO:0098809	nitrite reductase activity	24508	0	0.78		
GO:0051427	hormone receptor binding	24420	0	0.74		
GO:0031176	endo-1,4-beta-xylanase activity	24420	0	0.80		
GO:0016248	channel inhibitor activity	24377	0	0.69		
GO:0008200	ion channel inhibitor activity	24320	0	0.76		
GO:0017080	sodium channel regulator activity	23696	0	0.72		
GO:0017154	semaphorin receptor activity	23677	0	0.67		
GO:0050524	coenzyme-B sulfoethylthiotransferase activity	23651	0	0.62		
GO:0016985	mannan endo-1,4-beta-mannosidase activity	23160	0	0.92		
GO:0016824	hydrolase activity, acting on acid halide bonds	22939	0	0.88		
GO:0019120	hydrolase activity, acting on acid halide bonds, in C-halide compounds	22930	0	0.89		
GO:0005164	tumor necrosis factor receptor binding	22834	0	0.49		
GO:0016708	oxidoreductase activity, acting on paired donors, with incorporation or reductio...	22266	0	0.88		
GO:0004483	mRNA (nucleoside-2'-O-)-methyltransferase activity	21306	0	0.84		
GO:0005501	retinoid binding	21122	0	0.79		
GO:0005246	calcium channel regulator activity	20540	0	0.74		
GO:0003785	actin monomer binding	20314	0	0.51		
GO:0001540	amyloid-beta binding	20232	0	0.69		
GO:0048306	calcium-dependent protein binding	19589	0	0.55		
GO:0002039	p53 binding	19438	0	0.85		
GO:0005104	fibroblast growth factor receptor binding	18920	0	0.54		
GO:0034618	arginine binding	18834	0	0.64		
GO:0016972	thiol oxidase activity	17606	0	0.60		
GO:0035254	glutamate receptor binding	17506	0	0.80		
GO:0045735	nutrient reservoir activity	16951	2	0.81	0.66	
GO:1900750	oligopeptide binding	15092	0	0.88		
GO:0043295	glutathione binding	15045	0	0.89		
GO:0048487	beta-tubulin binding	14904	0	0.79		
GO:0009036	type II site-specific deoxyribonuclease activity	14686	0	0.86		
GO:0010427	abscisic acid binding	14290	0	0.67		
GO:0005537	mannose binding	13983	0	0.72		
GO:0015026	coreceptor activity	12746	0	0.35		
GO:0097718	disordered domain specific binding	12588	0	0.60		
GO:0030337	DNA polymerase processivity factor activity	11448	0	0.85		
GO:0050421	nitrite reductase (NO-forming) activity	11402	3	0.82	0.72	
GO:0015643	toxic substance binding	10829	0	0.85		
GO:0031720	haptoglobin binding	10250	0	0.67		
GO:0052716	hydroquinone:oxygen oxidoreductase activity	9876	0	0.77		
GO:0019871	sodium channel inhibitor activity	8600	0	0.63		
GO:0008135	translation factor activity, RNA binding	8319	1	0.52		
GO:0080030	methyl indole-3-acetate esterase activity	7849	0	0.95		
GO:0034987	immunoglobulin receptor binding	5373	0	0.64		
GO:0048020	CCR chemokine receptor binding	4975	0	0.46		
GO:0019865	immunoglobulin binding	4897	0	0.87		
GO:0030597	RNA glycosylase activity	3696	0	0.40		
GO:0030598	rRNA N-glycosylase activity	3633	0	0.50		
GO:0009374	biotin binding	3138	0	0.61		
GO:0016018	cyclosporin A binding	2759	19	0.63	0.53	0.61 (0.55)
GO:0032052	bile acid binding	2328	0	0.91		
GO:0070404	NADH binding	2238	0	0.93		
GO:0016918	retinal binding	2113	0	0.60		
GO:0016894	endonuclease activity, active with either ribo- or deoxyribonucleic acids and pr...	1605	0	0.66		
GO:0001883	purine nucleoside binding	848	0	0.90		
GO:0005527	macrolide binding	264	0	0.86		
GO:1903981	enterobactin binding	106	0	0.86		
GO:0032550	purine ribonucleoside binding	4	0	0.88		

bioRxiv preprint doi: https://doi.org/10.1101/2025.09.03.672144; this version posted September 4, 2025. The copyright holder for this preprint (which was not certified by peer review) is the author/funder, who has granted bioRxiv a license to display the preprint in perpetuity. It is made available under aCC-BY 4.0 International license.

Table S2: Additional results on active site structural alignments. For each of 45 GO terms, we randomly select up to 5 successful designs and, for each design, highlight one of the aligned AFDB / UniProt structures. We report the all-atom active site RMSD, the % of matching residue identities within 5 Å of the active site, the overall sequence identity, and the active site residues annoted in UniProtKB.

Term	Sample	AFDB / UniProt ID	Active site RMSD	5 Å ID	Seq. ID	Active site residues
GO:0003988	1	P07871	0.05	66.67	48.67	C123,C408
	2	Q7A2W9	0.16	63.64	43.0	C86,H338
GO:0004029	1	A0A3Q7PKI4	0.13	80.95	45.33	E210,C244
	2	A0A1L7TDK2	0.16	70.59	42.0	E228,C262
	3	A0A7W3NA22	0.2	90.48	54.33	E254,C288
	4	A0A2S5TF45	0.18	61.11	46.67	E221,C255
	5	A0A7X3WQ03	0.23	52.63	44.67	E214,C248
GO:0004030	1	A0A2D9E9P2	0.17	64.71	43.0	E190,C224
	2	P52476	0.28	68.75	46.67	E279,C313
	3	A6UQD0	0.24	66.67	42.33	E240,C274
	4	B8M9E2	0.41	61.11	39.0	E228,C262
	5	A0A7V9U005	0.15	72.22	57.33	E260,C294
GO:0004032	1	O13848	0.28	57.14	39.44	Y54,H109
	2	Q07551	0.13	45.0	41.67	Y64,H122
	3	Q07551	0.25	54.17	36.0	Y64,H122
	4	O13848	0.21	61.9	40.49	Y54,H109
	5	O14088	0.22	52.17	36.73	Y50,H111
GO:0004033	1	O14088	0.17	41.67	36.36	Y50,H111
	2	O14088	0.16	54.55	39.64	Y50,H111
	3	O13848	0.37	56.52	41.2	Y54,H109
	4	Q76L36	0.42	60.87	41.67	Y63,H125
	5	O14088	0.31	41.67	40.36	Y50,H111
GO:0004177	1	A0A0Q4DGT6	0.49	66.67	51.67	K256,R330
GO:0004190	1	A0A6P7MYT9	0.25	40.91	38.0	I139,T325
	2	A0A1L9WSA4	0.29	66.67	43.67	D82,D279
	3	A0A0D9RBZ8	0.29	72.22	45.0	D97,D295
	4	A0A319CTT4	0.19	66.67	35.33	D50,D252
	5	B8YJG5	0.16	65.22	38.0	D108,D291
GO:0004197	1	Q9GL24	0.11	81.82	46.33	C138,H277,N300
	2	Q10991	0.17	85.29	51.61	C25,H163,N184
	3	P09648	0.17	70.59	49.08	C25,H165,N185
GO:0004252	1	U9V2T3	0.15	58.62	39.06	D18,H50,S209
	2	A0A7V7QN85	0.16	66.67	38.0	D29,H61,S244
	3	A0A1I6AYN1	0.27	75.0	50.67	D178,H217,S434
	4	A0A1I5DDV9	0.15	77.42	49.33	D62,H110,S292
	5	A0A7X2J0A2	0.17	79.41	44.33	D137,H174,S352
GO:0004312	1	A0A6L7X5E9	0.22	54.17	41.33	S128,H233
	2	A0A535J0M4	0.46	45.83	39.0	S103,H212
	3	A0A3M1LXU7	0.48	47.83	39.33	S94,H201
	4	A0A2E6YIR2	0.3	60.0	37.0	S103,H208
	5	A0A2M8ETE7	0.2	42.86	33.0	S114,H219
GO:0004314	1	A0A352PU99	0.31	26.92	35.33	S91,H196
	2	A0A496AKM2	0.2	57.69	38.67	S96,H204
	3	A0A2J0KPU7	0.18	53.85	38.0	S145,H250
	4	Q5F4X7	0.2	53.85	37.0	S90,H199
	5	A0A1G3UH18	0.21	65.38	31.33	S92,H202
GO:0004315	1	P9WQD9	0.63	37.93	44.67	C171,H311,H345
	2	P0AAI7	0.24	56.67	43.0	C164,H304,H341
GO:0004525	1	A0A0F2Q590	0.14	56.52	30.29	D54,E126
	2	A0A535S225	0.05	56.52	34.91	D54,E124
	3	A0A2D6TAR6	0.16	56.52	34.03	D49,E121
GO:0004536	1	A0A351R9X6	0.31	51.52	29.69	Y104,D145,H246

bioRxiv preprint doi: https://doi.org/10.1101/2025.09.03.672144; this version posted September 4, 2025. The copyright holder for this preprint (which was not certified by peer review) is the author/funder, who has granted bioRxiv a license to display the preprint in perpetuity. It is made available under aCC-BY 4.0 International license.

Term	Sample	AFDB / UniProt ID	Active site RMSD	5 Å ID	Seq. ID	Active site residues
GO:0004550	1	A0A2J7QC69	0.32	60.71	39.67	H162,H316
	2	A0A803K9Z7	0.27	57.14	40.0	H212,H361
	3	D8TIX5	0.4	65.52	40.67	H203,H354
	4	A0A2J7QC53	0.31	60.71	41.33	H231,H385
	5	A0A2J7QC66	0.4	48.15	39.67	H208,H362
GO:0004620	1	P16233	0.21	50.0	36.0	S169,D193,H280
GO:0004725	1	A0A6M8FQ69	0.27	21.43	35.06	C7,R13,D123
GO:0004807	1	A0A840WL33	0.26	81.82	34.57	H93,E163
GO:0004843	1	Q8LAM0	0.31	28.57	38.67	C32,H310
	2	G5E8G2	0.09	66.67	41.67	C60,H307
	3	A0A7D8YLP6	0.06	71.43	50.0	C311,H741
	4	A0A0J8UXP0	0.28	38.1	48.0	C226,H657
	5	O57429	0.21	55.56	42.0	C28,H309
GO:0008080	1	A0A2E8FZC6	0.13	48.0	43.31	E109,Y121
	2	A0A7V7MUE6	0.14	50.0	48.57	E97,Y109
	3	A0A2W1KFP4	0.13	53.85	43.04	E103,Y115
	4	A0A411WMN2	0.24	37.04	46.58	E103,Y115
	5	A0A0U3AA44	0.21	48.0	43.23	E110,Y122
GO:0008106	1	A0A2K6B8V5	0.72	3.7	36.67	E210,C244
	2	O13848	0.19	54.55	41.2	Y54,H109
	3	O13848	0.36	59.09	42.25	Y54,H109
	4	A0A2K5NL52	0.55	3.57	39.67	E210,C244
	5	P30838	0.76	10.0	35.67	E210,C244
GO:0008234	1	P05993	0.15	68.18	51.04	H31,N58
	2	P09648	0.17	74.19	54.59	C25,H165,N185
	3	P83443	0.22	64.71	42.25	C26,H159,N176
	4	P83443	0.17	70.97	46.95	C26,H159,N176
	5	A5YVK8	0.2	76.47	51.63	C14,H146,N162
GO:0015035	1	A0A520C1H0	0.08	66.67	57.32	C7,C10
	2	A0A662FLU1	0.15	87.5	66.67	C30,C33
	3	A0A3D4FEA4	0.21	73.33	58.54	C7,C10
	4	A0A2K5CLF8	0.07	57.14	61.46	C23,C26
	5	A0A7Y2VGP6	0.1	66.67	56.47	C11,C14
GO:0015036	1	A0A182YSM0	0.07	66.67	63.95	C11,C14
	2	A0A1F9KUL7	0.1	73.33	55.56	C32,C35
	3	A0A7V3TX07	0.11	68.75	54.63	C32,C35
	4	A0A550H2S0	0.1	62.5	63.16	C20,C23
	5	A0A3D4FEA4	0.1	80.0	63.41	C7,C10
GO:0016298	1	A0A851Y268	0.23	51.61	36.67	S168,D194,H279
GO:0016407	1	A0A4P7JQ37	0.24	60.0	45.33	E107,Y119
	2	A0A3B9NVM9	0.16	41.67	48.63	E103,Y115
	3	A0A2W4VE46	0.23	61.54	48.67	E105,Y117
	4	A0A0P6VE99	0.76	50.0	45.62	E111,Y123
	5	A0A654L334	0.26	46.15	41.72	E99,Y111
GO:0016408	1	Q7A1P9	0.15	61.9	43.33	C86,H338
GO:0016410	1	D4XNE3	0.2	76.0	44.44	E99,Y111
	2	A0A6N6RS47	0.72	36.0	44.59	E104,Y116
	3	A0A2L1WHT7	0.25	52.0	48.67	E105,Y116
	4	A0A6N1N5Z4	0.19	64.0	42.76	E99,Y111
	5	A0A7C5L4R9	0.65	33.33	41.96	E97,Y109
GO:0016419	1	A0A3B0J3W0	0.18	53.85	37.0	S92,H201
	2	A0A077PZH2	0.17	46.43	41.67	S92,H201
	3	A0A0K2ART5	0.24	61.54	32.67	S89,H193
	4	A0A416WG28	0.27	53.85	30.67	S87,H198
	5	A0A3D3W9N5	0.28	46.15	35.0	S90,H195

bioRxiv preprint doi: https://doi.org/10.1101/2025.09.03.672144; this version posted September 4, 2025. The copyright holder for this preprint (which was not certified by peer review) is the author/funder, who has granted bioRxiv a license to display the preprint in perpetuity. It is made available under aCC-BY 4.0 International license.

Term	Sample	AFDB / UniProt ID	Active site RMSD	5 Å ID	Seq. ID	Active site residues
GO:0016420	1	A0A2D4TSP9	0.08	57.14	36.0	S90,H198
	2	A0A1C6NL35	0.11	56.0	36.67	S88,H193
	3	A0A536FEV0	0.27	48.15	43.0	S114,H221
	4	H2IQE4	0.3	52.0	23.33	S86,H195
	5	A0A101C5L5	0.22	46.15	33.67	S87,H195
GO:0016620	1	A0A7C7GGH8	0.14	66.67	31.0	E161,C195
	2	A0A3D0RY98	0.12	58.82	40.67	E232,C266
	3	A0A511J2Y8	0.16	66.67	46.33	E253,C287
	4	A0A1M5N6Y7	0.22	61.54	48.33	E233,C267
	5	A0A1R3FFM7	0.21	70.37	41.33	E217,C251
GO:0016667	1	A0A522QSK5	0.27	10.53	51.82	C32,C35
	2	A0A352HRW1	0.04	62.5	59.3	C7,C10
	3	A0A3D4FEA4	0.08	47.06	54.88	C7,C10
	4	A0A352PK77	0.1	80.0	51.92	C30,C33
	5	A0A3C0KDU2	0.04	50.0	70.15	C34,C37
GO:0016855	1	A0A256BFD5	0.28	19.05	30.43	C78,C187
GO:0016864	1	Q0E0I1	0.09	90.0	39.67	C75,C78
GO:0016884	1	E0NNP0	0.21	64.52	44.33	K69,S144,S168
GO:0030060	1	K0J107	0.28	55.0	40.33	D173,H200
	2	K0J107	0.23	76.19	40.33	D173,H200
	3	K0J107	0.36	52.38	40.0	D173,H200
	4	K0J107	0.32	63.64	39.0	D173,H200
GO:0032296	1	A0A149VUG9	0.21	50.0	37.83	D47,E119
	2	Q2NB81	0.18	50.0	39.01	D48,E119
GO:0033818	1	A0A535CXZ2	0.24	65.62	44.0	C129,H260,N290
	2	A0A0J9FDV5	0.25	54.55	32.33	C116,H256,N286
	3	A0A2V5ZBS8	0.59	33.33	35.67	C127,H267,N297
	4	A0A2V3W4S1	0.53	37.5	32.0	C112,H237,N267
	5	A0A6I3ZG68	0.61	35.29	40.33	C122,H258,N289
GO:0047661	1	A0A252DYH1	0.23	25.0	26.62	C91,C200
	2	A0A0R2B670	0.36	56.0	40.21	C73,C184
	3	A0A5M8R285	0.26	52.0	25.28	C75,C188
	4	R7IW21	0.58	31.82	25.9	C79,C190
GO:0051920	1	A0A3P1CVP9	0.12	26.67	33.67	C158,C161
	2	A0A243RTW8	0.15	53.33	41.57	C131,C134
GO:0052689	1	A0A4Y8L3A9	0.32	32.26	28.0	S93,D192,H222
	2	A0A2E9H3J6	0.25	41.94	32.55	S81,D206,H234
	3	A0A4R0ZJD2	0.22	35.71	29.48	S98,D194,H223
	4	A0A7X1TML9	0.35	44.83	30.33	S186,D277,H306
	5	A0A523I713	0.32	44.83	27.0	S169,D255,H285
GO:0052745	1	Q3ZCK3	0.13	47.62	34.0	D51,T122
	2	Q9Z0S1	0.64	33.33	32.0	D51,T122
GO:0070001	1	A0A1Y1ZC50	0.09	60.87	33.33	D17,D198
	2	A0A818CU57	0.12	69.57	48.0	D304,D485
	3	A0A3B5Y713	0.49	59.09	35.33	D126,D349
	4	F9XJD5	0.2	56.52	32.0	D91,D273
	5	A0A667INK2	0.12	60.87	35.67	D94,D281
GO:0070008	1	Q8L9Y0	0.22	67.86	48.67	S185,D395,H447
	2	Q9CAU2	0.49	46.67	32.33	S183,D363,H416
	3	Q869Q8	0.34	58.62	38.33	S233,D414,H474
GO:0101005	1	A6NNY8	0.14	71.43	42.33	C87,H380
	2	Q9LEW0	0.14	71.43	45.33	C186,H491
	3	P62068	0.15	65.0	42.67	C44,H313
	4	Q52KZ6	0.13	66.67	43.33	C48,H317
	5	Q9FPS3	0.17	61.11	45.67	C206,H510
